# Targeting the IL-6–Yap–Snail signalling axis in synovial fibroblasts ameliorates inflammatory arthritis

**DOI:** 10.1136/annrheumdis-2021-220875

**Published:** 2021-11-29

**Authors:** Rebecca A Symons, Fabio Colella, Fraser L Collins, Alexandra J Rafipay, Karolina Kania, Jessica J McClure, Nathan White, Iain Cunningham, Sadaf Ashraf, Elizabeth Hay, Kevin S Mackenzie, Kenneth A Howard, Anna H K Riemen, Antonio Manzo, Susan M Clark, Anke J Roelofs, Cosimo De Bari

**Affiliations:** 1 Arthritis and Regenerative Medicine Laboratory, Aberdeen Centre for Arthritis and Musculoskeletal Health, Institute of Medical Sciences, University of Aberdeen, Aberdeen, UK; 2 Microscopy and Histology Core Facility, Institute of Medical Sciences, University of Aberdeen, Aberdeen, UK; 3 Interdisciplinary Nanoscience Center (iNANO), Department of Molecular Biology and Genetics, Aarhus University, Aarhus, Denmark; 4 Rheumatology and Translational Immunology Research Laboratories (LaRIT), Division of Rheumatology, IRCCS Policlinico San Matteo Foundation, University of Pavia, Pavia, Italy

**Keywords:** arthritis, experimental, rheumatoid, fibroblasts, inflammation

## Abstract

**Objective:**

We aimed to understand the role of the transcriptional co-factor Yes-associated protein (Yap) in the molecular pathway underpinning the pathogenic transformation of synovial fibroblasts (SF) in rheumatoid arthritis (RA) to become invasive and cause joint destruction.

**Methods:**

Synovium from patients with RA and mice with antigen-induced arthritis (AIA) was analysed by immunostaining and qRT-PCR. SF were targeted using *Pdgfrα-CreER* and *Gdf5-Cre* mice, crossed with fluorescent reporters for cell tracing and *Yap-flox* mice for conditional *Yap* ablation. Fibroblast phenotypes were analysed by flow cytometry, and arthritis severity was assessed by histology. Yap activation was detected using Yap–Tead reporter cells and Yap–Snail interaction by proximity ligation assay. SF invasiveness was analysed using matrigel-coated transwells.

**Results:**

Yap, its binding partner Snail and downstream target connective tissue growth factor were upregulated in hyperplastic human RA and in mouse AIA synovium, with Yap detected in SF but not macrophages. Lineage tracing showed polyclonal expansion of *Pdgfrα*-expressing SF during AIA, with predominant expansion of the *Gdf5*-lineage SF subpopulation descending from the embryonic joint interzone. *Gdf5*-lineage SF showed increased expression of *Yap* and adopted an erosive phenotype (podoplanin+Thy-1 cell surface antigen−), invading cartilage and bone. Conditional ablation of *Yap* in *Gdf5*-lineage cells or *Pdgfrα*-expressing fibroblasts ameliorated AIA. Interleukin (IL)-6, but not tumour necrosis factor alpha (TNF-α) or IL-1β, Jak-dependently activated Yap and induced Yap–Snail interaction. SF invasiveness induced by IL-6 stimulation or Snail overexpression was prevented by Yap knockdown, showing a critical role for Yap in SF transformation in RA.

**Conclusions:**

Our findings uncover the IL-6–Yap–Snail signalling axis in pathogenic SF in inflammatory arthritis.

**Key messages**

**What is already known about this subject?**

- Synovial fibroblasts (SF) are key drivers of rheumatoid arthritis (RA) pathogenesis; however, the SF lineages involved and the molecular mechanisms underpinning their pathogenic transformation are incompletely understood.

**What does this study add?**

- This study shows that *Gdf5*-lineage SF, defined by their ontogenic derivation from the joint interzone, are the key pathogenic SF in inflammatory arthritis. Mechanistically, interleukin (IL)-6 signals via Jak to activate Yes-associated protein (Yap), which forms a complex with Snail to drive the invasiveness of SF, and selective targeting of Yap in *Gdf5*-lineage SF ameliorates inflammatory arthritis.

**How might this impact on clinical practice or future developments?**

- This study supports the targeting of IL-6 and Jak in RA, not only for immunosuppression but also to directly control the Yap–Snail-mediated pathogenic behaviour of SF.

## Introduction

Rheumatoid arthritis (RA) is a common immune-mediated chronic inflammatory disease causing joint damage and deformities. Current treatment consists of synthetic and biological disease-modifying antirheumatic drugs aimed at systemic immunosuppression. Nonetheless, some patients fail to respond to treatments, and joint damage progression can still occur despite clinical remission.^1^

A hallmark of RA pathology is synovitis, causing the synovium to thicken and form a pannus that invades cartilage and bone, driven by pathogenic transformation and proliferation of fibroblasts with infiltration of inflammatory/immune cells. A recent study reported two distinct subsets of synovial fibroblasts (SF), immune effector fibroblasts expressing podoplanin (Pdpn) and Thy-1 cell surface antigen (Thy1) found in the sub-lining synovial tissue and promoting inflammation, and destructive Pdpn+Thy1− fibroblasts in the lining layer mediating cartilage and bone damage.^2^

Cell lineage tracing studies have revealed that the adult synovium consists of ontogenetically diverse fibroblast subsets. One subset, the *Gdf5*-lineage cells, descend from the *Gdf5*-expressing joint interzone cells in the embryo.^3–5^ Previously, we have shown that these *Gdf5*-lineage cells include the SF in the lining as well as a subset of SF in the sublining.^5 6^ The remaining SF are of unknown embryonic origin. It is not known how the ontogenetically defined SF subsets relate to the subsets identified by Pdpn and Thy1 expression.

The synovial pannus is a tumour-like structure resulting in part from uncontrolled fibroblast expansion. Increased activity of the transcriptional cofactor Yes-associated protein (Yap) is known to cause tissue overgrowth in multiple tissues and organs through stimulating cell proliferation.^7–13^ In addition, Yap promotes cell motility and invasion in cancer cells.^14–16^ We demonstrated that Yap promotes proliferation of fibroblast-like mesenchymal cells^17^ and that increased Yap activity in *Gdf5*-lineage cells underpins synovial lining hyperplasia following acute joint surface injury in mice.^5^

The role of Yap in immune-mediated inflammatory arthritis and the signalling mechanisms that link Yap activity to the pathogenic transformation of SF await clarification. A reduced severity in the K/BxN serum-transfer arthritis model in mice was reported after treatment with the small molecule verteporfin,^18^ a non-specific inhibitor of Yap activity. Although the mechanism was not investigated, it was suggested that the beneficial effect was through reduced Yap activity in SF.^18^ However, it cannot be excluded that the effects of verteporfin were Yap-independent.^19 20^ Furthermore, verteporfin was reported to ameliorate antigen-induced arthritis (AIA) in rabbits by inducing apoptosis of inflammatory cells.^21^

Here, we show that Yap is highly expressed by SF in both human RA and mouse AIA and that conditional genetic ablation of Yap in SF ameliorates AIA. Mechanistically, Yap is Jak-dependently activated by interleukin (IL)-6, a key inflammatory cytokine in RA, and forms a complex with the transcription factor Snail to drive the SF invasive phenotype. Our findings identify the IL-6–Yap–Snail signalling axis as a fibroblast-specific therapeutic target in RA synovitis.

## Methods

Materials and methods are available in the online supplemental information file.

10.1136/annrheumdis-2021-220875.supp1 Supplementary data

## Results

### Yap is upregulated in human rheumatoid and mouse immune-mediated synovitis

**Figure 1:**
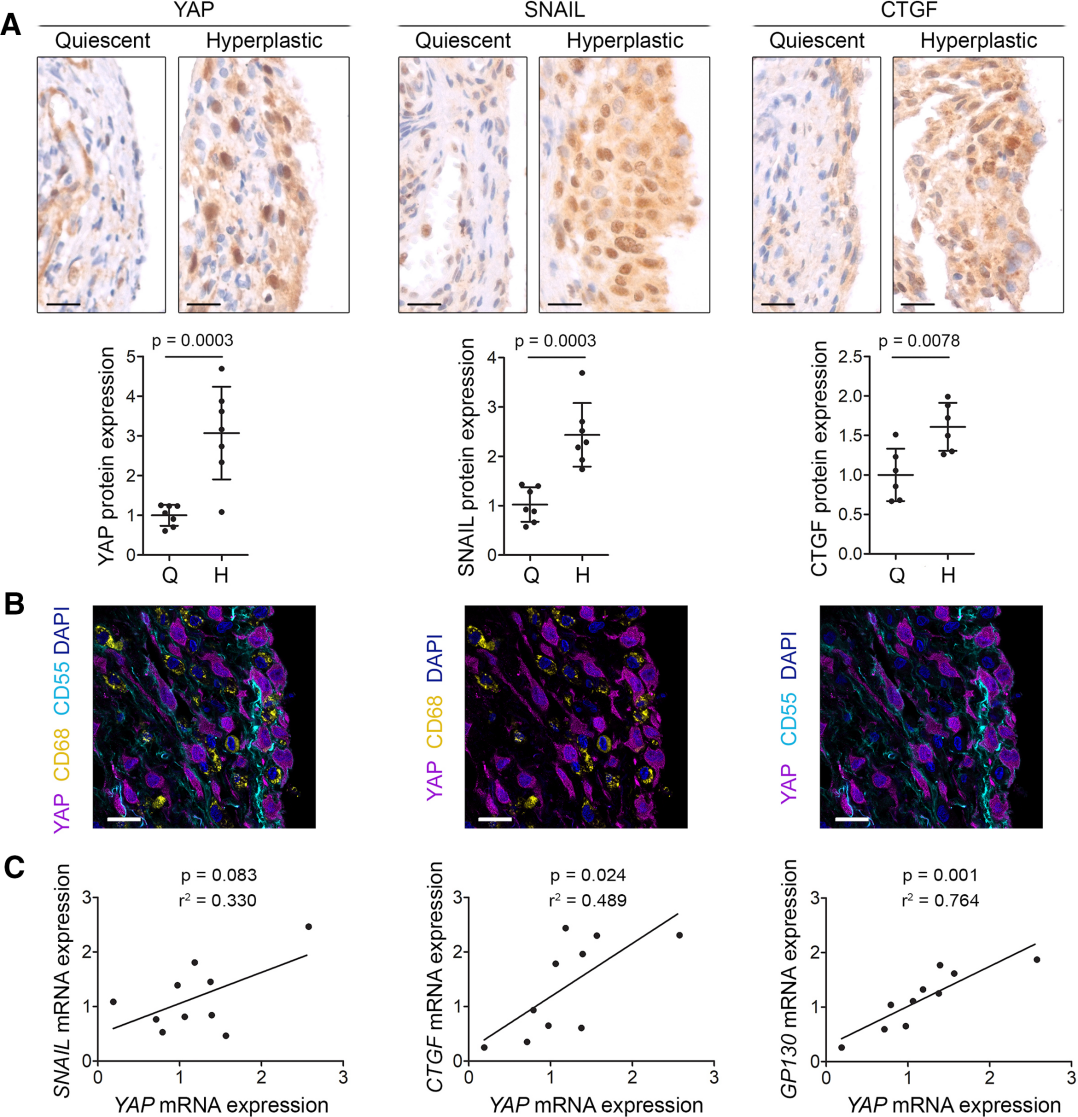
YAP, SNAIL and CTGF are upregulated in hyperplastic rheumatoid arthritis synovium. (A) Immunohistochemical detection of YAP, SNAIL and CTGF in quiescent and hyperplastic areas of human RA synovium (YAP n=7, SNAIL n=7, and CTGF n=6). Haematoxylin counterstain is shown in blue. Scale bars: 20 µm. For isotype negative control stainings, see online supplemental figure 10a–c. Graphs indicate protein expression in quiescent (Q) and hyperplastic (H) synovium based on immunohistochemistry (IHC) staining intensity, with lines and error bars indicating mean±SD (n=6–7). P values indicate statistical significance using an unpaired two-tailed t-test. (B) Expression of YAP (magenta), the SF marker CD55 (cyan) and the macrophage marker CD68 (yellow) in human RA synovium (n=4). DAPI (4′,6-diamidino-2-phenylindole) nuclear counterstain is shown in blue. The same image is shown three times with different channels overlaid, for clarity. Scale bars: 20 µm. For isotype negative control stainings, see online supplemental figure 10d. (C) Correlation between *YAP* gene expression and expression of *SNAIL*, *CTGF* or *GP130* in RA synovial biopsies (n=10) as determined by qRT-PCR. P values indicate results of Pearson’s correlation test and R^2^ values the square of the correlation coefficient. CTGF, connective tissue growth factor; RA, rheumatoid arthritis; SF, synovial fibroblast; YAP, Yes-associated protein.

YAP was upregulated in the hyperplastic compared with quiescent lining of human RA synovium (p=0.0003, figure 1A), and this was accompanied by upregulation of the transcription factor SNAIL (p=0.0003), and the YAP and SNAIL downstream target gene connective tissue growth factor (CTGF) (p=0.0078, figure 1A), a known pathogenic effector in RA.^22^ YAP was expressed by CD55+ fibroblasts and not by CD68+ macrophages (figure 1B). *YAP* mRNA expression levels correlated non-significantly with the expression of *SNAIL* (p=0.083) and significantly with the expression of YAP downstream targets *CTGF* (p=0.024) and *GP130* (p=0.001, figure 1C), a transmembrane protein required for IL-6 signalling.^23^

**Figure 2:**
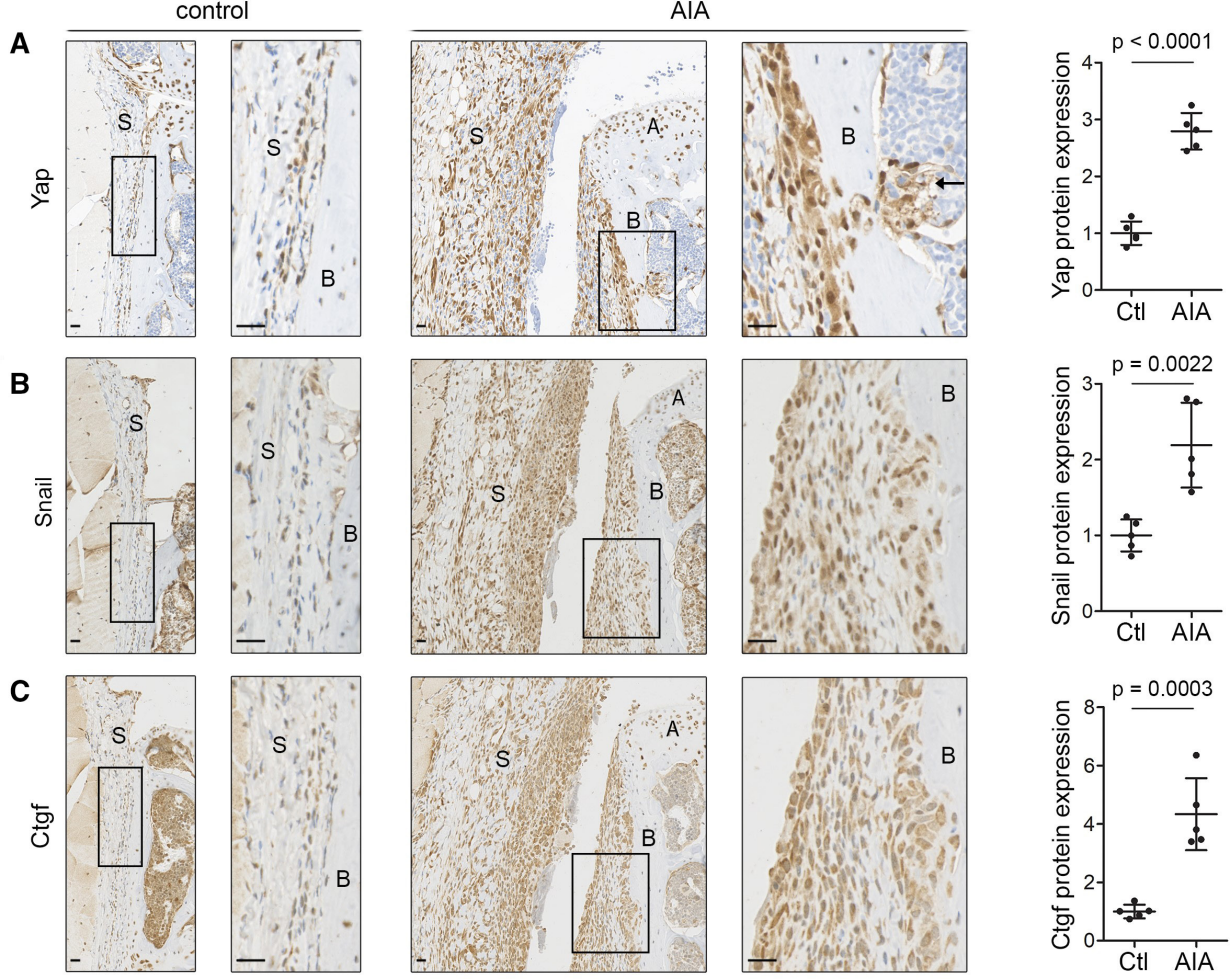
Yap, Snail and Ctgf are upregulated in inflammatory arthritis in mice. (A–C) Immunohistochemical detection of Yap (A), Snail (B) and Ctgf (C) in mouse synovium 6 days after AIA induction (n=5; 2 male mice, 3 female mice, 11–13 weeks). Contralateral knee served as control. Haematoxylin counterstain is shown in blue. Boxed areas on the left are shown at higher magnification on the right. Arrow (A) indicates Yap-expressing cells penetrating through the bone into the underlying marrow space. Scale bars: 20 µm. For isotype negative control stainings, see online supplemental figure 10f–h. Graphs indicate protein expression in synovium based on IHC staining intensity, with lines and error bars indicating mean±SD (n=5). P values indicate statistical significance using an unpaired two-tailed t-test. AIA, antigen-induced arthritis; A, articular cartilage; B, bone; Ctgf, connective tissue growth factor; S, synovium; Yap, Yes-associated protein.

Analysis of mouse AIA synovium confirmed the upregulation of Yap (p<0.0001, figure 2A), Snail (p=0.0022, figure 2B) and Ctgf (p=0.0003, figure 2C) during synovitis. High Yap expression was observed in fibroblast-like cells throughout the synovium and along the periosteal surface, extending into the underlying marrow space at sites of erosive damage (figure 2A and online supplemental figure 1a).

### *Gdf5*-lineage Yap-expressing SF are predominant in arthritis

**Figure 3:**
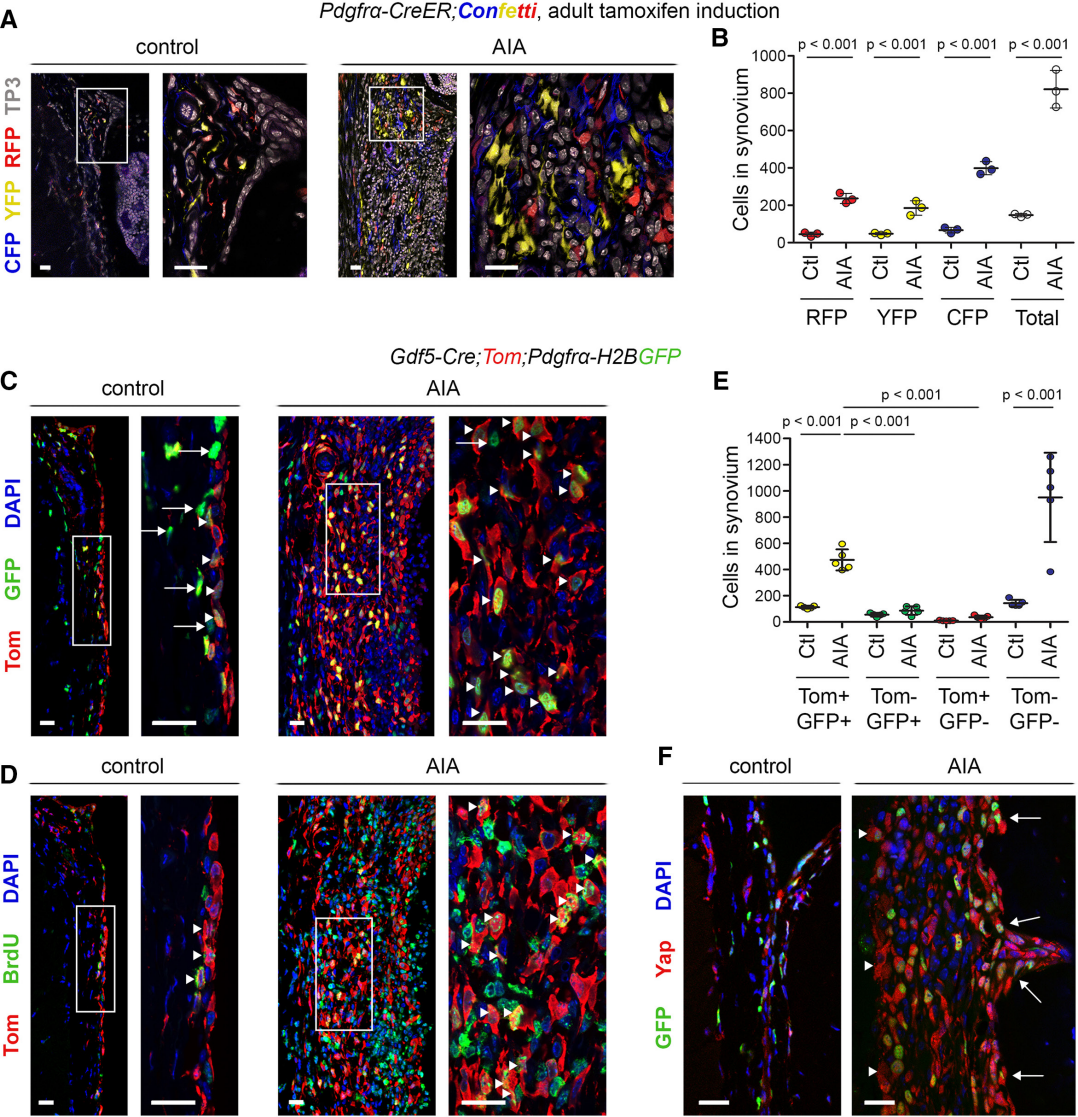
*Pdgfrα*-expressing *Gdf5*-lineage cells expand during inflammatory arthritis. (A, B) Detection of *Pdgfrα*-traced cells, marked by expression of CFP, YFP or RFP, in synovium of *Pdgfrα-CreER;Confetti* mice induced with tamoxifen from 8 weeks of age prior to AIA induction and analysed after 6 days of AIA (n=3; 3 male mice, 14–15 weeks). Contralateral knee served as control. (A) Cells labelled by CFP (blue), YFP (yellow) and RFP (red) in synovium. TO-PRO-3 (TP3) nuclear counterstain is shown in grey. (B) Numbers of CFP, YFP and RFP-labelled cells per mm length of synovium. P values indicate statistical significance based on two-way ANOVA with Tukey’s post-test after log transformation (n=3). (C–F) *Gdf5-Cre;Tom;Pdgfrα-H2BGFP* mice 6 days after AIA induction (n=5; 2 male mice, 3 female mice, 11–13 weeks). Mice received bromodeoxyuridine (BrdU) to label proliferating cells from arthritis induction until the end. (C, D) Expansion of Tom-labelled *Gdf5*-lineage cells (red) in synovium during AIA, co-staining for (C) GFP (green), indicative of *Pdgfrα* expression (arrowheads indicate Tom+GFP+ cells; arrows indicate Tom-GFP+ cells), or (D) the BrdU proliferation label (green; arrowheads indicate Tom+BrdU+ cells). Consecutive tissue sections are shown. For isotype negative control stainings, see online supplemental figure 10i, j. (E) Numbers of Tom-labelled and GFP-labelled cells per mm length of synovium. P values indicate statistical significance based on two-way ANOVA with Tukey’s post-test after log-transformation (n=5). (F) Detection of Yap (red) in GFP-expressing cells (green) in synovial lining (arrowheads) and along the periosteal surface (arrows). Scale bars: 20 µm. For isotype negative control stainings, see online supplemental figure 10k. Lines and error bars on all graphs indicate mean±SD. AIA, antigen-induced arthritis; ANOVA, analysis of variance.

Next, we used genetic cell-labelling and tracing models to map fibroblast populations in synovitis. To trace individual fibroblasts, we used *Pdgfrα-CreER;R26-Confetti* mice (see online supplemental table 1 for transgenic mouse lines used in the study), in which tamoxifen administration activates CreER in cells expressing the pan-fibroblast marker *Pdgfrα*, resulting in stochastic expression of one of four fluorescent proteins.^6^ Analysis of cyan fluorescent protein (CFP), yellow fluorescent protein (YFP) and red fluorescent protein (RFP) expression (green fluorescent protein (GFP) was rarely detected and was omitted from analysis) 6 days after AIA induction revealed extensive expansion of *Pdgfrα*-traced cells in synovium. Multiple small clusters of monochromatic cells were interspersed throughout the synovium, indicating polyclonal cell expansion (figure 3A, B).

We previously showed that a subset of *Pdgfrα*-expressing cells in the adult synovium originates from the *Gdf5*-expressing embryonic joint interzone.^5 6^ These *Gdf5*-lineage SF are mostly found in the synovial lining but also include a subset of the SF in the sublining.^5^ To determine the involvement of the *Gdf5*-lineage SF in synovitis, we induced AIA in *Gdf5-Cre;Tom;Pdgfrα-H2BGFP* mice and administered BrdU to label proliferating cells. In these mice, Cre is expressed and permanently switches on Tom expression in cells of the joint interzone and all their progeny, while Cre is not active in the adult knee.^24–26^ In addition, *Pdgfrα*-expressing fibroblasts are identified by long-lived nuclear GFP. In control knees, *Gdf5*-lineage SF (ie, Tom+GFP+ cells) were mostly quiescent and located predominantly in the synovial lining, while other SF (ie, Tom-GFP+ cells) were found throughout the synovium (figure 3C, D). In contrast, *Gdf5*-lineage SF extensively proliferated and infiltrated the entire synovium in the AIA knee (figure 3C, D), increasing 4.2±0.6-fold (mean±SD, n=5, p<0.001) and constituting the dominant SF lineage in the inflamed synovium (figure 3E). A concomitant increase in the unlabelled cell population (ie, Tom−GFP− cells) reflects the infiltration and expansion of immune cells during synovitis (figure 3E). GFP and Yap co-detection confirmed Yap expression by SF in both the synovial lining and along the periosteal surface (figure 3F), and Yap expression along the bone surface, including at sites of erosion, correlated with the presence of *Gdf5*-lineage cells (online supplemental figure 1).

**Figure 4:**
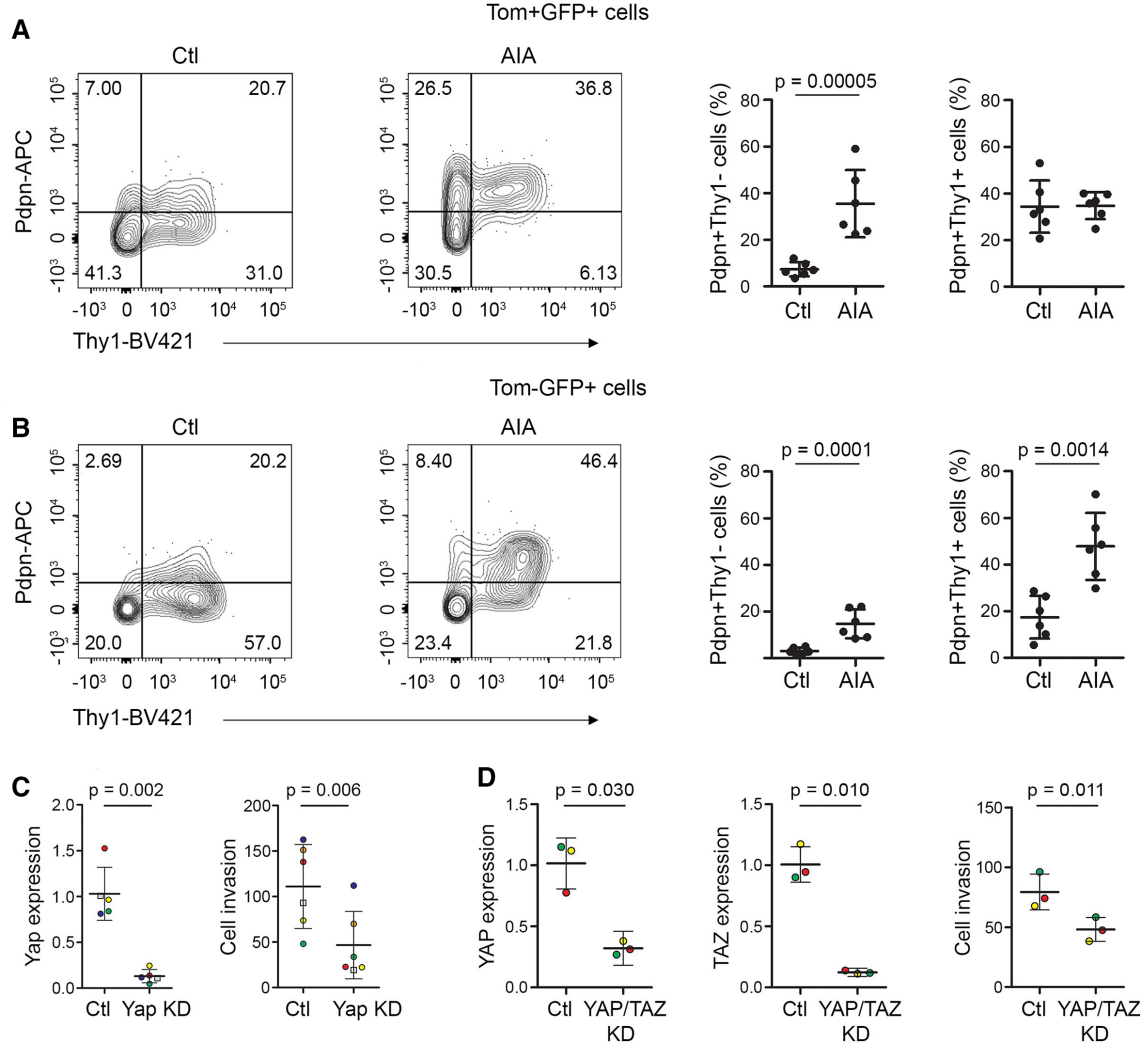
Phenotypic analysis of SF in inflammatory arthritis. (A, B) Freshly isolated cells from knees of adult *Gdf5-Cre;Tom;Pdgfrα-H2BGFP* mice 6 days after AIA induction (n=6; 5 male mice and one female mouse, 11–14 weeks, pooled data from two experiments) were analysed by flow cytometry for the expression of Pdpn and Thy1 within (A) the *Gdf5*-lineage *Pdgfrα*-expressing cells (Tom+GFP+) and (B) the remaining *Pdgfrα*-expressing cells (Tom-GFP+). The contralateral knees served as controls. Graphs show the percentage of cells expressing Pdpn with or without coexpression of Thy1. P values indicate statistical significance based on unpaired two-tailed t-test after log transformation. For gating strategy and staining controls, see online supplemental figure 2. (C) Yap KD decreased AIA-SF invasiveness through matrigel in a transwell assay. Dots are colour-coded to indicate different experiments (n=5 for Yap expression, n=6 for cell invasion) using cells from three different mice for DsiRNA#1 (circles), and from a fourth mouse for DsiRNA#2 (squares). P values indicate results of two-tailed paired Student’s t-test. (D) YAP/TAZ KD decreased human RA-SF invasiveness through matrigel in a transwell assay. Dots are colour-coded to indicate independent experiments using cells from different donors (n=3). P values indicate results of two-tailed paired Student’s t-test. AIA, antigen-induced arthritis; KD, knockdown; Pdpn, podoplanin; RA, rheumatoid arthritis; SF, synovial fibroblast; Thy1, Thy-1 cell surface antigen.

A recent study reported Pdpn+Thy1+ SF to be immunomodulatory and Pdpn+Thy1− SF to be erosive in inflammatory arthritis.^3^ Hence, we investigated the expression of Pdpn and Thy1 in the ontogenetically defined SF subsets. While *Gdf5*-lineage SF included both Pdpn+Thy1+ and Pdpn+Thy1− phenotypic subsets, we observed a striking increase in the percentage of *Gdf5*-lineage SF with a Pdpn+Thy1− (erosive) phenotype during AIA, as compared with control knees (p=0.00005, figure 4A and online supplemental figure 2). In contrast, the other SF adopted primarily a Pdpn+Thy1+ (immunomodulatory) phenotype in response to AIA (p=0.0014, figure 4B and online supplemental figure 2).

To determine whether Yap controls the capacity of SF to remodel and invade extracellular matrix, we used a matrigel-coated Boyden transwell assay.^27^ DsiRNA-mediated knockdown of Yap in SF from AIA mice reduced their invasive ability (p=0.006, figure 4C). Notably, knockdown of the Yap paralog, Taz, did not affect mouse SF invasiveness (online supplemental figure 3). In human RA-SF, reduced invasiveness was observed after simultaneous knockdown of YAP and TAZ (p=0.011, figure 4D).

Altogether, these data show extensive proliferation and expansion of Yap-expressing SF throughout the synovium in AIA and reveal the *Gdf5*-lineage SF to be the predominant erosive SF subset in immune-mediated synovitis, with Yap being required for SF invasiveness in vitro.

### Conditional ablation of *Yap* in SF ameliorates inflammatory arthritis

**Figure 5:**
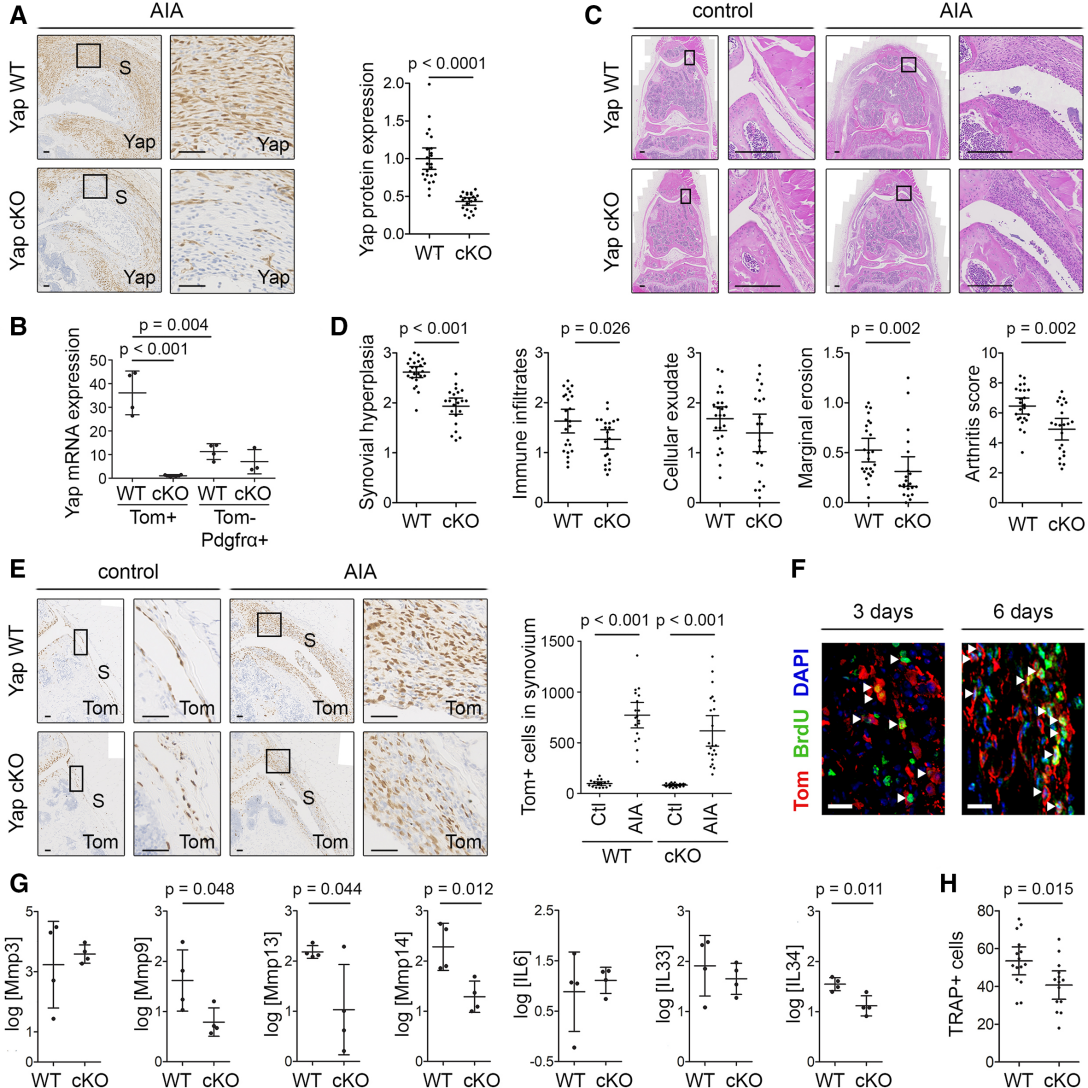
Ablation of *Yap* in *Gdf5*-lineage cells reduces inflammatory arthritis severity. AIA was induced in one knee of *Yap* WT or *Yap* cKO mice, with contralateral knee serving as control, and mice were analysed after 3 or 6 days (F), 7 days (B, G) or 9 days (A, C–E, H). See online supplemental table 2 for mouse genotypes, sex, age and exclusions. (A) Yap expression in inflamed synovium detected by IHC. Images show mice with highest arthritis score in their group. Scale bars: 50 µm. P value: unpaired two-tailed t-test after log transformation (WT: n=24; cKO: n=22; five experiments). (B) *Yap* mRNA expression detected by qRT-PCR in fluorescence-activated cell sorting (FACS)-sorted SF from AIA knees of *Yap* WT (n=4) and *Yap* cKO mice (n=3–4; 1 *Yap* cKO Tom-Pdgfrα+ sorted sample was excluded from analysis due to very low cell yield). P values indicate results of two-way analysis of variance with Tukey’s post-test after log transformation. (C, D) H&E staining and histological scoring of arthritis severity. Images show matched control and AIA knees from mice with arthritis scores (WT: 6.33; cKO: 4.66) close to their group average. Scale bars: 200 µm. P values: Mann-Whitney U test (WT: n=24; cKO: n=22; five experiments). (E) Tom+ *Gdf5*-lineage cells in synovium detected by IHC. Images are from the same mice as in (C). Scale bars: 50 µm. Graph shows Tom+ cells per millimetre length of synovium. P values: Kruskal-Wallis test with Dunn’s post-test (WT: n=18; cKO: n=22; five experiments). (F) Immunofluorescent detection of BrdU (green) in Tom+ *Gdf5*-lineage cells (red) in the synovium of *Yap* cKO mice at 3 (n=5) or 6 days (n=3) after AIA induction. Scale bars: 20 µm. (G) Expression of Mmps and cytokines detected by qRT-PCR in *Gdf5*-lineage cells FACS-sorted from knees of *Yap* WT (n=4) or *Yap* cKO (n=4) mice. P values: unpaired two-tailed t-test after log transformation. (H) TRAP+ cells along the medial and lateral femoral periosteal surface of female mice. P value: unpaired two-tailed t-test (WT: n=15; cKO: n=14; five experiments). Boxed areas in images on the left are shown at higher magnification on the right. For isotype negative control stainings, see online supplemental figure 10e, f, j. For FACS strategy, see online supplemental figure 4. Data on graphs are shown as mean±95% CI (A, D, E, H) or mean±SD (B, G). AIA, antigen-induced arthritis; S, synovium; SF, synovial fibroblast; WT, wild type; Yap, Yes-associated protein.

To determine the role of Yap in *Gdf5*-lineage SF in the pathophysiology of inflammatory arthritis, we conditionally ablated *Yap* in *Gdf5*-lineage cells and induced AIA. A Cre-inducible *Tom* reporter was crossed into the model, allowing detection of *Yap* conditional KO (cKO) target cells. Immunostaining of synovium showed a significant decrease in Yap expression in the cKO mice (p<0.0001, figure 5A). The efficiency of *Yap* cKO was further confirmed at mRNA level by qRT-PCR of sorted cells (p<0.001), which moreover revealed a significantly higher expression of *Yap* in the *Gdf5*-lineage SF compared with other SF after 7 days of AIA (p=0.004; figure 5B and online supplemental figure 4), further supporting a key role for Yap in the *Gdf5*-lineage AIA-SF.

Nine days after arthritis induction, we observed significant decreases in synovial lining hyperplasia (p<0.001), immune infiltrates in synovium (p=0.026) and erosions at the joint margins (p=0.002), and a non-significant trend towards decreased cellular exudate, resulting in an overall arthritis score of 4.9 (95% CI 4.2 to 5.6, n=22) in the cKO mice, compared with 6.5 (95% CI 5.9 to 7.0, n=24) in *Yap* WT mice (p=0.002; figure 5C, D). Despite the decrease in arthritis severity in vivo and diminished cell proliferation after *Yap* KO in vitro (p=0.023) (online supplemental figure 5), extensive expansion of *Gdf5*-lineage cells in synovium during AIA was observed in both *Yap* WT (p<0.001) and *Yap* cKO mice (p<0.001, figure 5E). Accordingly, using BrdU labelling, we detected proliferation of *Gdf5*-lineage cells throughout the synovium in *Yap* cKO mice at 3 and 6 days after AIA induction (figure 5F). These data indicate that Yap is largely dispensable for SF proliferation but required for SF-mediated disease activity in AIA.

Next, we investigated the effects of conditional *Yap* ablation on the expression of key catabolic and inflammatory mediators by qRT-PCR analysis of Tom+ *Gdf5*-lineage SF purified from AIA mice. Compared with *Gdf5*-lineage SF of *Yap* WT mice, the *Gdf5*-lineage SF from *Yap* cKO mice displayed reduced expression levels of critical enzymes mediating SF invasiveness, including Mmp9 (p=0.048), Mmp13 (p=0.044) and Mmp14 (p=0.012),^28–30^ as well as the inflammatory cytokine IL-34 (p=0.011) (figure 5G). These data point to a role for Yap in mediating critical arthritogenic effects of SF that contribute to erosive damage and inflammation in AIA.

In RA, erosive damage to bone is caused by increased osteoclastic bone resorption, driven in part by pro-osteoclastogenic factors produced by the SF.^31^ Staining for the osteoclast marker TRAP revealed a decreased number of TRAP+ cells along the femoral periosteal surface in female *Yap* cKO mice compared with *Yap* WT controls after AIA (p=0.015; figure 5H and online supplemental figure 6), while no significant difference was observed in the number of TRAP+ cells at endosteal surfaces of the femoral epiphysis (online supplemental figure 6). Accordingly, microCT analysis showed no difference in the trabecular bone loss in the tibial epiphysis between *Yap* WT and *Yap* cKO mice in response to AIA (online supplemental figure 7). Together, these data suggest a role for Yap in SF in stimulating local osteoclast development in female mice.

**Figure 6:**
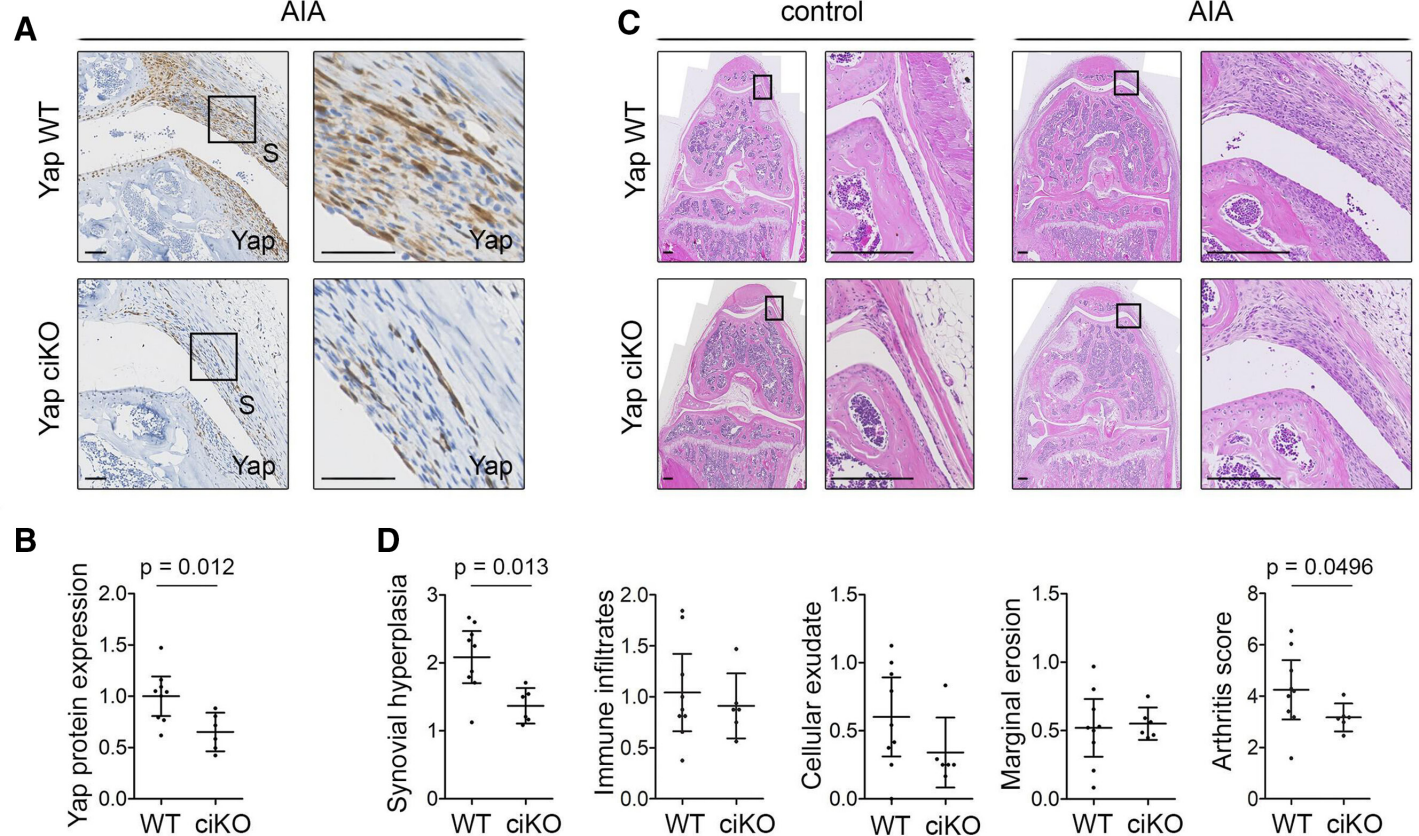
Inducible ablation of *Yap* in *Pdgfrα*-expressing fibroblasts reduces inflammatory arthritis severity. Adult *Yap^fl/fl^* (Yap WT) or *Pdgfrα-CreER;Yap^fl/fl^* (Yap ciKO) mice received tamoxifen to activate Cre and KO *Yap*, prior to induction of AIA in one knee, with the contralateral knee serving as control. Histological analysis was performed 9 days after arthritis induction. See online supplemental table 3 for mouse genotypes, sex and age. (A) Yap expression in the inflamed synovium detected by IHC with haematoxylin counterstain. Histological images were selected from the same mice as shown in panel (C). Boxed areas in images on the left are shown at higher magnification on the right. Scale bars: 50 µm. For isotype negative control staining, see online supplemental figure 10f. (B) Yap expression in synovium, based on IHC staining intensity. P value indicates result of two-tailed unpaired t-test (WT: n=9; ciKO n=6; pooled data from two experiments). (C) Synovitis and erosive damage in AIA knees detected by H&E staining. Images show matched control and AIA knees from female mice in the same experiment with arthritis scores (WT: 5.00; ciKO: 2.99) close to the average of their respective groups. Boxed areas in images on the left are shown at higher magnification on the right. Scale bars: 200 µm. (D) Histological assessment of severity of synovial hyperplasia, immune infiltrates, cellular exudate and marginal erosions (all on scale 0–3), and overall arthritis severity (scale 0–12). P values indicate results of Mann-Whitney U test (WT: n=9; ciKO: n=6, pooled data from two experiments). Lines and error bars on all graphs indicate mean±95% CI. AIA, antigen-induced arthritis; S, synovium; WT, wild type; Yap, Yes-associated protein.

Next, we sought to validate these data using a conditional tamoxifen-inducible (ci) KO model in which *Yap* is ablated in the SF of the adult knee, using *Pdgfrα-CreER* as driver. Immunodetection of Yap in tissue sections confirmed *Yap* ciKO (figure 6A, B), although with lower efficiency than *Gdf5-Cre*-driven *Yap* cKO (figure 5A). Nonetheless, we again observed a decrease in synovial lining hyperplasia (p=0.013) and arthritis score (p=0.0496) compared with *Yap* WT mice (figure 6C, D).

Together, these findings indicate that Yap has crucial functions in pathogenic SF to promote inflammation and joint destruction, and demonstrate amelioration following its selective targeting in SF in inflammatory arthritis.

### IL-6 activates Yap through Jak and induces Yap–Snail interaction in SF to drive their invasiveness

**Figure 7:**
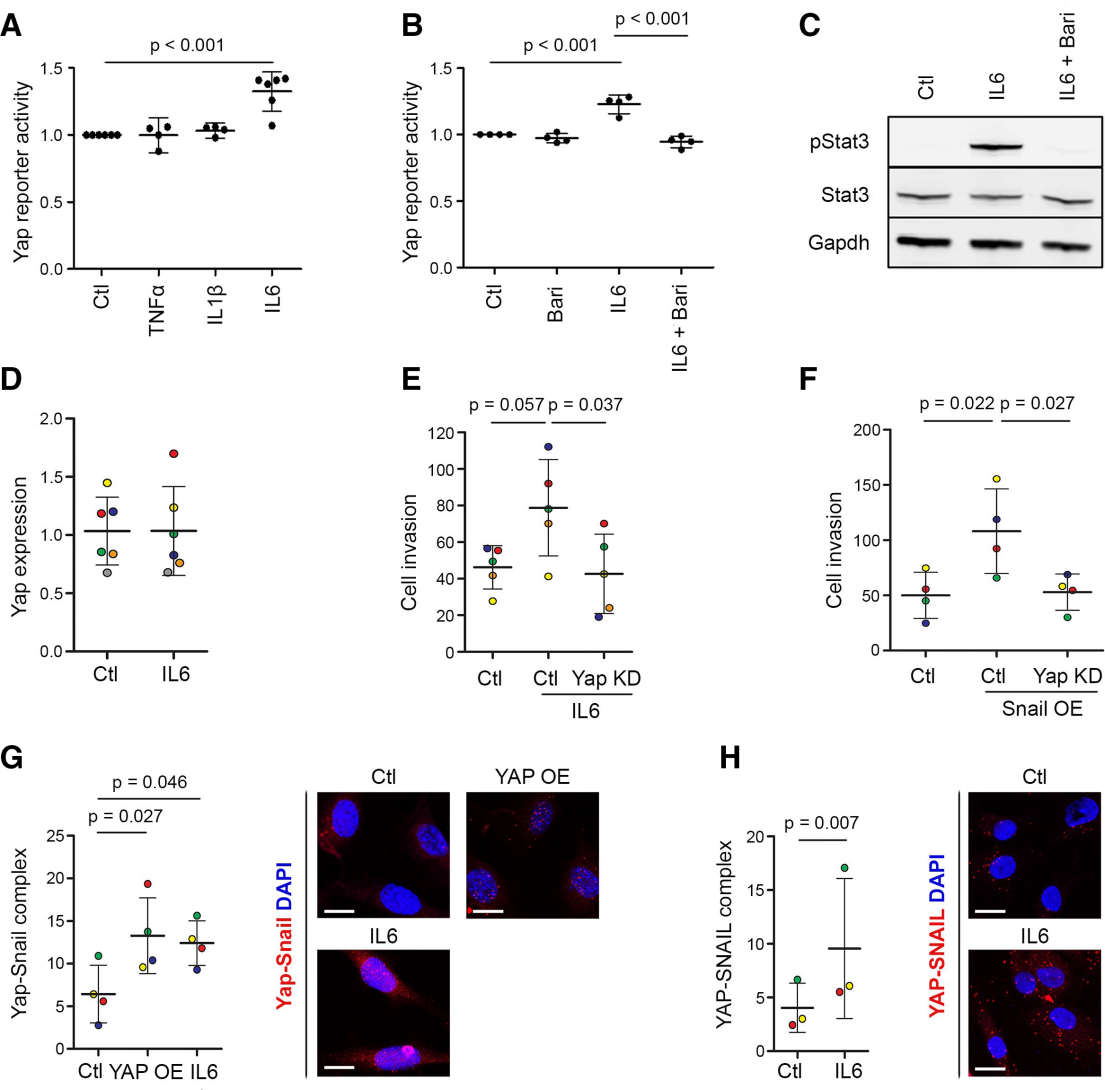
IL-6 activates Yap through Jak and drives SF invasion by stimulating Yap–Snail interaction. (A) IL-6 activates Yap. Yap–Tead GFP reporter cells were stimulated with 10 ng/mL TNF-α, 10 ng/mL IL-1β or 20 ng/mL IL-6/sIL6R, and GFP expression was quantified by flow cytometry (n=4–6 experiments). P value: one-way repeated measures ANOVA with Tukey’s post-test, performed on data before normalisation. (B) The Jak inhibitor baricitinib prevents IL-6-induced Yap activation. Yap–Tead GFP reporter cells were treated with IL-6/sIL6R (20 ng/mL) and baricitinib (1 or 2 µM) for 48 hours under vehicle-controlled conditions, and GFP expression was quantified by flow cytometry (n=4 experiments). P values: one-way repeated measures ANOVA with Tukey’s post-test, performed on data before normalisation. (C) Baricitinib prevents IL-6/sIL6R-induced Stat3 phosphorylation. Yap–Tead GFP reporter cells were pretreated for 1 hour with baricitinib (10 µM) and then treated with IL-6/sIL6R (140 ng/mL) for 30 min, as indicated, under vehicle-controlled conditions. Data are representative of n=3 experiments. See online supplemental figure 11 for uncropped Western blot images. (D) IL-6/sIL6R treatment (10 ng/mL) does not affect Yap mRNA expression in AIA-SF after 24 hours. (E) Yap KD prevents the increased AIA-SF invasiveness after IL-6/sIL6R treatment (10 ng/mL) for 48 hours. Dots are colour-coded to indicate five independent experiments using cells from four different mice. P values: one-way repeated measures ANOVA with Tukey’s post-test. (F) Yap KD prevents the increased invasion of AIA-SF induced by Snail overexpression. Dots are colour-coded to indicate four independent experiments using cells from three different mice. P values: one-way repeated measures ANOVA with Tukey’s post-test. (G) Treatment with IL-6/sIL6R (5 ng/mL) for 4 hours increased Yap–Snail complex formation in mouse SF, detected using proximity ligation assay. Transfection with constitutively active YAP-S127A was used as a positive control. Dots are colour-coded to indicate independent experiments using cells from different mice (n=4). P values: repeated measures one-way ANOVA with Dunnet’s post-test. Scale bars: 20 µm. (H) Treatment with IL-6/sIL6R (5 ng/mL) for 4 hours increased YAP–SNAIL complex formation in human SF, detected using proximity ligation assay. Dots are colour-coded to indicate independent experiments using cells from different donors (n=3). P value: two-tailed paired Student’s t-test. Scale bars: 20 µm. Lines and error bars on all graphs indicate mean±SD. ANOVA, analysis of variance; IL, interleukin; KD, knockdown; OE, overexpression; SF, synovial fibroblast; Yap, Yes-associated protein.

To assess the ability of key inflammatory cytokines to activate Yap, we used a Yap–Tead reporter construct,^32^ modified to drive GFP expression following stable lentiviral transduction (online supplemental figure 8). We detected selective Yap activation by IL-6/sIL6R (p<0.001), while TNF-α or IL-1β had no effect (figure 7A). Baricitinib, a selective Jak1/2 inhibitor, prevented the IL-6/sIL6R-induced activation of Yap (p<0.001, figure 7B), as well as phosphorylation of Stat3 (figure 7C), indicating that IL-6-induced Yap activation requires Jak signalling. IL-6/sIL6R stimulation did not increase *Yap* mRNA expression at least after 24 hours (figure 7D), indicating that its effect may be mediated through post-transcriptional mechanisms. Yap knockdown prevented IL-6/sIL6R-induced invasiveness of normal mouse SF (figure 7E), showing that IL-6 signals via Yap to promote invasion. An IL-6/sIL6R complex was used in these experiments, since SF do not express the IL-6R (online supplemental figure 9) and rely on sIL6R, normally produced by immune cells, to activate IL-6 trans-signalling via gp130.^33^

Since the transcription factor Snail, previously reported to be involved in TNF-α-mediated SF activation in RA,^34^ was upregulated alongside Yap during RA and AIA synovitis (figures 1A and 2B), we investigated the functional relationship between Snail and Yap. Snail overexpression increased SF invasiveness (p=0.022), and this was prevented by simultaneous knockdown of Yap (p=0.027, figure 7F), demonstrating that Snail requires Yap to drive SF invasion. Using a proximity ligation assay, we found that treatment of mouse SF or human RA-SF with IL-6/sIL6R induced the formation of Yap–Snail complexes, similar to what is seen after Yap overexpression in mouse SF (figure 7G, H). Taken together, these data indicate that IL-6 trans-signalling in SF activates Yap through Jak and increases Yap–Snail interaction to promote invasiveness.

## Discussion

SF are key cells in RA that sustain inflammation and induce tissue damage, but the molecular mechanisms underlying these functional characteristics remain to be fully elucidated. Here, we report that ablation of *Yap* in SF ameliorates immune-mediated inflammatory arthritis in vivo and show that IL-6 increases Yap activity through Jak signalling and promotes interaction of Yap and Snail to drive the pathogenic behaviour of SF.

In a previous study using the K/BxN serum-transfer mouse model of inflammatory arthritis and mice engrafted with cartilage and RA-SF, treatment with the non-specific Yap inhibitor verteporfin was shown to reduce arthritis severity and cartilage invasion by RA-SF, respectively.^18^ However, verteporfin exhibits Yap-independent cytostatic and cytotoxic effects and was previously shown to ameliorate AIA by inducing immune cell apoptosis.^19–21^ Additionally, Yap is highly expressed by endothelium and known to regulate endothelial cell proliferation, migration and survival.^35^ Indeed, Yap was recently reported to mediate synovial angiogenesis in AIA mice, with verteporfin shown to reduce angiogenesis and synovitis.^35^ Here, we unequivocally show, by conditional ablation of *Yap* in SF using two different mouse models, a specific role for Yap in driving the pathological behaviour of SF in inflammatory arthritis. The recent demonstration of additional roles for Yap in promoting synovial angiogenesis further supports the possible therapeutic benefits of targeting Yap in RA synovitis.^35^

We previously reported that increased Yap activity in *Gdf5*-lineage SF drives synovial lining hyperplasia in a traumatic joint surface injury model in mice.^5^ In the present study, we show that ablation of *Yap* in *Gdf5-*lineage SF reduced not only synovial lining hyperplasia but also immune infiltration and erosive damage in inflammatory arthritis. Decreased synovial lining hyperplasia in AIA was also observed when Yap was knocked out in fibroblasts in adult mice, showing this is not a result of developmental defects resulting from Yap KO.

Although the upstream regulators of Yap activity are likely to be context-dependent, we uncover a hitherto unappreciated molecular link between Jak-mediated IL-6 signalling, pivotal in RA pathogenesis, and the Yap-mediated invasive SF phenotype. While IL-6 treatment of SF enhanced invasive behaviour, Yap silencing completely prevented the IL-6-induced SF invasion. JAK inhibition with baricitinib was shown to abrogate cytokine-induced invasive behaviour of SF.^36^ Together, this suggests that JAK inhibitors such as baricitinib could ameliorate RA disease in part by modulating YAP activity in SF.

SF, which do not express the IL-6R, undergo pro-inflammatory sIL6R-mediated trans-signalling, facilitated by cell surface expression of GP130.^33^ We showed a strong correlation between *YAP* and *GP130* expression in human RA synovium, in accordance with evidence that YAP upregulates *GP130* expression in an autoregulatory loop,^23^ indicating that YAP plays a role in amplifying IL-6 signalling and maintaining invasiveness in RA-SF. YAP activation by IL-6 has also been reported in human colorectal cancer cells.^37^ It is therefore plausible that the IL-6-GP130-YAP pathway becomes activated in multiple disorders, possibly as a general response to abnormal contexts jeopardising organ/tissue homeostasis.

Snail was previously reported to be highly expressed in RA synovium and SF, and its lentiviral vector-mediated silencing in the joint ameliorated collagen-induced arthritis in rats.^34^ We describe the formation of a complex between Yap and Snail in SF from AIA mice and patients with RA. This complex was reported to occur in skeletal stem cells to modulate their physiological functions of self-renewal and lineage commitment,^38^ suggesting cell-specific and context-dependent functions of the Yap–Snail complex. Silencing of Yap was sufficient to prevent the invasive phenotype induced by Snail overexpression, demonstrating a requirement for active Yap to mediate SF invasiveness. Intriguingly, while Snail was reported to regulate the TNF-α-mediated activation of SF,^34^ in our study activation of Yap occurred downstream of IL-6 only, not TNF-α. Altogether, these findings configure a synergistic cooperation of the inflammatory cytokine network in RA to activate SF and transform them into destructive cells via inducing Yap–Snail interaction.

A recent study reported functionally distinct fibroblast subsets in RA synovium, with destructive fibroblasts restricted to the synovial lining layer and immune effector fibroblasts located in the synovial sub-lining.^2^ In our study, we show that while in normal knee joint synovium the *Gdf5*-lineage SF were largely confined to the synovial lining, as previously reported,^5^ in AIA synovitis the *Gdf5*-lineage SF underwent extensive proliferation and expansion throughout the synovium, indicating a derangement of the anatomical segregation of fibroblast lineages during synovitis. Our findings show the *Gdf5*-lineage SF to become erosive and support a role for Yap in the destructive SF, possibly through modulation of MMP expression.^28–30^ The reduced immune cell infiltrates in the synovium of *Yap* cKO mice with AIA suggests that the *Gdf5*-lineage also includes immune effector SF and that Yap may be needed in these cells to sustain inflammation. Indeed, *Gdf5*-lineage SF from *Yap* cKO AIA mice displayed reduced expression levels of IL-34, an inflammatory cytokine produced by RA-SF able to support osteoclastogenesis^39^ and reported to be associated with synovitis severity in patients with RA.^40^

In summary, we report a novel IL-6-Jak-Yap–Snail signalling axis linking immune-mediated inflammation with pathogenic SF in RA. Our findings position Yap in RA-SF at the crossroads between the activated immune system and their destructive phenotype, and suggest Yap for SF-targeted therapy in RA.

## Data Availability

Data are available upon reasonable request. All data relevant to the study are included in the article or uploaded as supplemental information.

## References

[1] Smolen JS , Aletaha D , Barton A , et al . Rheumatoid arthritis. Nat Rev Dis Primers 2018;4:18001. 10.1038/nrdp.2018.1 29417936

[2] Croft AP , Campos J , Jansen K , et al . Distinct fibroblast subsets drive inflammation and damage in arthritis. Nature 2019;570:246–51. 10.1038/s41586-019-1263-7 31142839 PMC6690841

[3] Rountree RB , Schoor M , Chen H , et al . BMP receptor signaling is required for postnatal maintenance of articular cartilage. PLoS Biol 2004;2:e355. 10.1371/journal.pbio.0020355 15492776 PMC523229

[4] Koyama E , Shibukawa Y , Nagayama M , et al . A distinct cohort of progenitor cells participates in synovial joint and articular cartilage formation during mouse limb skeletogenesis. Dev Biol 2008;316:62–73. 10.1016/j.ydbio.2008.01.012 18295755 PMC2373417

[5] Roelofs AJ , Zupan J , Riemen AHK , et al . Joint morphogenetic cells in the adult mammalian synovium. Nat Commun 2017;8:15040. 10.1038/ncomms15040 28508891 PMC5493527

[6] Roelofs AJ , Kania K , Rafipay AJ , et al . Identification of the skeletal progenitor cells forming osteophytes in osteoarthritis. Ann Rheum Dis 2020;79:1625–34. 10.1136/annrheumdis-2020-218350 32963046 PMC8136618

[7] Dong J , Feldmann G , Huang J , et al . Elucidation of a universal size-control mechanism in Drosophila and mammals. Cell 2007;130:1120–33. 10.1016/j.cell.2007.07.019 17889654 PMC2666353

[8] Camargo FD , Gokhale S , Johnnidis JB , et al . YAP1 increases organ size and expands undifferentiated progenitor cells. Curr Biol 2007;17:2054–60. 10.1016/j.cub.2007.10.039 17980593

[9] Lu L , Li Y , Kim SM , et al . Hippo signaling is a potent in vivo growth and tumor suppressor pathway in the mammalian liver. Proc Natl Acad Sci U S A 2010;107:1437–42. 10.1073/pnas.0911427107 20080689 PMC2824398

[10] Schlegelmilch K , Mohseni M , Kirak O , et al . Yap1 acts downstream of α-catenin to control epidermal proliferation. Cell 2011;144:782–95. 10.1016/j.cell.2011.02.031 21376238 PMC3237196

[11] Zhou D , Zhang Y , Wu H , et al . Mst1 and MST2 protein kinases restrain intestinal stem cell proliferation and colonic tumorigenesis by inhibition of Yes-associated protein (YAP) overabundance. Proc Natl Acad Sci U S A 2011;108:E1312–20. 10.1073/pnas.1110428108 22042863 PMC3241824

[12] Cao X , Pfaff SL , Gage FH . YAP regulates neural progenitor cell number via the TEA domain transcription factor. Genes Dev 2008;22:3320–34. 10.1101/gad.1726608 19015275 PMC2600760

[13] Zhang H , Pasolli HA , Fuchs E . Yes-associated protein (YAP) transcriptional coactivator functions in balancing growth and differentiation in skin. Proc Natl Acad Sci U S A 2011;108:2270–5. 10.1073/pnas.1019603108 21262812 PMC3038759

[14] Zhang X , Yang L , Szeto P , et al . The Hippo pathway oncoprotein YAP promotes melanoma cell invasion and spontaneous metastasis. Oncogene 2020;39:5267–81. 10.1038/s41388-020-1362-9 32561850

[15] Illes B , Fuchs A , Gegenfurtner F , et al . Spatio-selective activation of nuclear translocation of YAP with light directs invasion of cancer cell spheroids. iScience 2021;24:102185. 10.1016/j.isci.2021.102185 33718837 PMC7921841

[16] Tremblay AM , Missiaglia E , Galli GG , et al . The Hippo transducer Yap1 transforms activated satellite cells and is a potent effector of embryonal rhabdomyosarcoma formation. Cancer Cell 2014;26:273–87. 10.1016/j.ccr.2014.05.029 25087979

[17] Karystinou A , Roelofs AJ , Neve A , et al . Yes-associated protein (YAP) is a negative regulator of chondrogenesis in mesenchymal stem cells. Arthritis Res Ther 2015;17:1–14. 10.1186/s13075-015-0639-9 26025096 PMC4449558

[18] Bottini A , Wu DJ , Ai R , et al . PTPN14 phosphatase and YAP promote TGFβ signalling in rheumatoid synoviocytes. Ann Rheum Dis 2019;78:600–9. 10.1136/annrheumdis-2018-213799 30808624 PMC7039277

[19] Zhang H , Ramakrishnan SK , Triner D , et al . Tumor-selective proteotoxicity of verteporfin inhibits colon cancer progression independently of YAP1. Sci Signal 2015;8:ra98. 10.1126/scisignal.aac5418 26443705 PMC4818013

[20] Dasari VR , Mazack V , Feng W , et al . Verteporfin exhibits YAP-independent anti-proliferative and cytotoxic effects in endometrial cancer cells. Oncotarget 2017;8:28628–40. 10.18632/oncotarget.15614 28404908 PMC5438678

[21] Ratkay LG , Chowdhary RK , Iamaroon A , et al . Amelioration of antigen-induced arthritis in rabbits by induction of apoptosis of inflammatory cells with local application of transdermal photodynamic therapy. Arthritis Rheum 1998;41:525–34. 10.1002/1529-0131(199803)41:3&lt;525::AID-ART19&gt;3.0.CO;2-I 9506581

[22] Nozawa K , Fujishiro M , Kawasaki M , et al . Inhibition of connective tissue growth factor ameliorates disease in a murine model of rheumatoid arthritis. Arthritis Rheum 2013;65:1477–86. 10.1002/art.37902 23436223

[23] Taniguchi K , Moroishi T , de Jong PR , et al . YAP-IL-6ST autoregulatory loop activated on APC loss controls colonic tumorigenesis. Proc Natl Acad Sci U S A 2017;114:1643–8. 10.1073/pnas.1620290114 28130546 PMC5320959

[24] Chen H , Capellini TD , Schoor M , et al . Heads, shoulders, Elbows, knees, and toes: modular GDF5 enhancers control different joints in the vertebrate skeleton. PLoS Genet 2016;12:e1006454. 10.1371/journal.pgen.1006454 27902701 PMC5130176

[25] Pregizer SK , Kiapour AM , Young M , et al . Impact of broad regulatory regions on Gdf5 expression and function in knee development and susceptibility to osteoarthritis. Ann Rheum Dis 2018;77:450. 10.1136/annrheumdis-2017-212475 29311146 PMC6338229

[26] Kania K , Colella F , Riemen AHK , et al . Regulation of Gdf5 expression in joint remodelling, repair and osteoarthritis. Sci Rep 2020;10:157. 10.1038/s41598-019-57011-8 31932746 PMC6957535

[27] Lee DM , Kiener HP , Agarwal SK , et al . Cadherin-11 in synovial lining formation and pathology in arthritis. Science 2007;315:1006–10. 10.1126/science.1137306 17255475

[28] Miller M-C , Manning HB , Jain A , et al . Membrane type 1 matrix metalloproteinase is a crucial promoter of synovial invasion in human rheumatoid arthritis. Arthritis Rheum 2009;60:686–97. 10.1002/art.24331 19248098 PMC2819053

[29] Jüngel A , Ospelt C , Lesch M , et al . Effect of the oral application of a highly selective MMP-13 inhibitor in three different animal models of rheumatoid arthritis. Ann Rheum Dis 2010;69:898–902. 10.1136/ard.2008.106021 19497915 PMC2925150

[30] Xue M , McKelvey K , Shen K , et al . Endogenous MMP-9 and not MMP-2 promotes rheumatoid synovial fibroblast survival, inflammation and cartilage degradation. Rheumatology 2014;53:2270–9. 10.1093/rheumatology/keu254 24982240

[31] Danks L , Komatsu N , Guerrini MM , et al . RANKL expressed on synovial fibroblasts is primarily responsible for bone erosions during joint inflammation. Ann Rheum Dis 2016;75:1187–95. 10.1136/annrheumdis-2014-207137 26025971

[32] Dupont S , Morsut L , Aragona M , et al . Role of YAP/TAZ in mechanotransduction. Nature 2011;474:179–83. 10.1038/nature10137 21654799

[33] Rose-John S . Il-6 trans-signaling via the soluble IL-6 receptor: importance for the pro-inflammatory activities of IL-6. Int J Biol Sci 2012;8:1237–47. 10.7150/ijbs.4989 23136552 PMC3491447

[34] Chen S-Y , Shiau A-L , Li Y-T , et al . Transcription factor snail regulates tumor necrosis factor α-mediated synovial fibroblast activation in the rheumatoid joint. Arthritis Rheumatol 2015;67:39–50. 10.1002/art.38899 25303734

[35] Chen Q , Fan K , Chen X , et al . Ezrin regulates synovial angiogenesis in rheumatoid arthritis through YAP and Akt signalling. J Cell Mol Med 2021;25:9378–89. 10.1111/jcmm.16877 34459110 PMC8500952

[36] Karonitsch T , Beckmann D , Dalwigk K , et al . Targeted inhibition of Janus kinases Abates interfon gamma-induced invasive behaviour of fibroblast-like synoviocytes. Rheumatology 2018;57:572–7. 10.1093/rheumatology/kex426 29228301

[37] Rosenbluh J , Nijhawan D , Cox AG , et al . β-Catenin-driven cancers require a Yap1 transcriptional complex for survival and tumorigenesis. Cell 2012;151:1457–73. 10.1016/j.cell.2012.11.026 23245941 PMC3530160

[38] Tang Y , Feinberg T , Keller ET . Snail/Slug-YAP/TAZ complexes control skeletal stem cell self- renewal and differentiation. Nat Cell Biol 2016;18:917–29. 27479603 10.1038/ncb3394 PMC5007193

[39] Hwang S-J , Choi B , Kang S-S , et al . Interleukin-34 produced by human fibroblast-like synovial cells in rheumatoid arthritis supports osteoclastogenesis. Arthritis Res Ther 2012;14:R14. 10.1186/ar3693 22264405 PMC3392804

[40] Chemel M , Le Goff B , Brion R , et al . Interleukin 34 expression is associated with synovitis severity in rheumatoid arthritis patients. Ann Rheum Dis 2012;71:150–4. 10.1136/annrheumdis-2011-200096 22039170 PMC3413617

