# Supplementary material for: Targeting the IL-6–Yap–Snail signalling axis in synovial fibroblasts ameliorates inflammatory arthritis
Source: Ann Rheum Dis. 2021 Nov 29;81(2):214–24. doi: 10.1136/annrheumdis-2021-220875 (PMC8762018; doi:10.1136/annrheumdis-2021-220875)
Supplement: Supplementary data [file annrheumdis-2021-220875supp001.pdf]

Supplementary Information

Targeting the IL6-Yap-Snail signalling axis in synovial fibroblasts ameliorates inflammatory arthritis

Rebecca Symons[1]*, Fabio Colella[1]*, Fraser L. Collins[1], Alexandra J. Rafipay[1], Karolina Kania[1], Jessica McClure[1], Nathan White[1], Iain Cunningham[1], Sadaf Ashraf[1], Elizabeth Hay[1], Kevin S. Mackenzie[2], Kenneth A. Howard[3], Anna H.K. Riemen[1], Antonio Manzo[4], Susan M. Clark[1], Anke J. Roelofs[1¶], Cosimo De Bari[1¶]

[1] Arthritis & Regenerative Medicine Laboratory, Aberdeen Centre for Arthritis and Musculoskeletal Health, Institute of Medical Sciences, University of Aberdeen, Aberdeen AB25 2ZD, United Kingdom;

[2] Microscopy and Histology Core Facility, Institute of Medical Sciences, University of Aberdeen, Aberdeen AB25 2ZD, United Kingdom;

[3] Interdisciplinary Nanoscience Center (iNANO), Department of Molecular Biology and Genetics, Aarhus University, Aarhus, Denmark;

[4] Rheumatology and Translational Immunology Research Laboratories (LaRIT), Division of Rheumatology, IRCCS Policlinico San Matteo Foundation/University of Pavia, Italy.

*¶ These authors contributed equally.

Correspondence: Cosimo De Bari, MD PhD FRCP, Institute of Medical Sciences, University of Aberdeen, Foresterhill, Aberdeen AB25 2ZD, UK. Tel: +44-1224-437477, E-mail: c.debari@abdn.ac.uk.

METHODS

Human tissue collection

Human synovial tissue samples were obtained from patients with a clinical diagnosis of RA after informed consent, under the auspices of the NHS Grampian Biorepository and IRCCS Policlinico San Matteo Foundation, either via ultrasound-guided synovial biopsy or during knee arthroplasty. Full patient information is provided in Supplementary Table 4.

Mice

Animal experimental protocols were approved by the UK Home Office and the Animal Welfare and Ethical Review Committee of the University of Aberdeen. All animal experiments were performed at the University of Aberdeen Medical Research Facility after a minimum acclimatisation period of 6 days. Experiments were designed to ensure that minimum numbers of mice were used to obtain biologically significant results. To study the effect of *Yap* ablation in SF on arthritis severity, we adopted a group-sequential design to minimise animal use. *Pdgfrα-H2BGFP*, *Pdgfrα-CreER*, *Gdf5-Cre*, Cre-inducible *R26-TdTomato* (*Tom*), Cre-inducible *R26-Confetti*, and *Yap*^{fl} mice (Supplementary Table 1) were crossed to generate mice for experiments. Genotyping was performed by PCR using DNA extracted from earclips, or from post-mortem tissue biopsies. For *Yap* KO experiments, genotypes are indicated in Supplementary Tables 2 and 3. All other transgenic mice were hemi/heterozygous for their respective transgenes. Genotypes were confirmed at the analysis stage by PCR using DNA extracted from tissue sections, detection of Yap by IHC, or detection of fluorescent protein expression. Mice whose genotype at the time of analysis did not correspond with the genotype determined *a priori* were either reassigned or excluded. *Gdf5-Cre;Tom* mice showing widespread (leaky) Cre-mediated recombination[1] were excluded *a priori* via detection of Tom expression in peripheral blood,[2] or at

the analysis stage. Mice were maintained on a 12:12 light-dark cycle with water and standard chow provided *ad libitum*. To induce Cre-mediated recombination, starting at 8-9 weeks of age, *Pdgfrα-CreER;Confetti* mice, or *Pdgfrα-CreER*;*Yap*$^{fl/fl}$ and genotype control mice, received 200 mg/kg tamoxifen dissolved in corn oil daily via oral gavage for 5 days, and 9 days later for a further 5 days. Fluorescent protein expression was negligible in the absence of tamoxifen treatment. The arthritis induction protocol was started 11-12 days after the last tamoxifen dose.

Antigen-induced arthritis (AIA)

Male and female mice, aged 8-14 weeks, were immunised with 200 µg methylated bovine serum albumin (mBSA, Sigma) in 200 µl Complete Freund's Adjuvant (Sigma) injected intradermally at 4 sites under isoflurane anaesthesia. Seven days later, arthritis was induced by intra-articular injection of 100 µg mBSA in 10 µl phosphate-buffered saline (PBS) into the right knee under isoflurane anaesthesia. The left knee (control) was injected with PBS only (Figs. 2-3) or not injected (Figs. 4-6). Paracetamol (200 mg/kg) mixed with soft food was provided for 3 days following arthritis induction (Figs. 4, 5). Mice were humanely killed between 3 and 9 days after AIA induction, and knees were collected for analysis. For *Yap* KO experiments, mice were age- and sex-matched to their genotype controls (Supplementary Tables 2 and 3). Arthritis induction was performed in no particular order by researchers blinded to genotypes, and mice were maintained in the same room. Randomisation was not applicable to this study since contralateral limbs served as controls, or group allocation was based on genotypes. For BrdU-labelling of proliferating cells, 2 mg BrdU was either injected intraperitoneally on day 2 after AIA induction and mice were killed 3 days post-AIA, or injected subcutaneously at the time of AIA induction followed by 1 mg/ml BrdU in drinking water until mice were killed at 6 days post-AIA.

Histological analyses

Mouse knees or human synovial tissue samples were fixed in paraformaldehyde, EDTA-decalcified as needed, and paraffin- or cryo-sectioned, as described.[2] For *Yap* KO experiments, DNA was extracted from paraffin tissue sections to confirm mouse genotypes by PCR. Immunohistochemistry (IHC) and immunofluorescence (IF) stainings were performed as described[3] using antibodies listed in Supplementary Table 5. To validate antibody stainings, donor-matched sections were stained, in parallel, with isotype negative control antibodies that were species-matched, concentration-matched, and matched for clonality to the antibody of interest. TRAP staining was performed using an Acid Phosphatase, Leukocyte (TRAP) kit (Sigma-Aldrich) according to manufacturer's protocol. Images were acquired on a Zeiss Axioscan Z1, Olympus upright microscope, or Zeiss LSM710 confocal microscope with ZEN software.

Image analysis

Zen 2.1 Lite software was used to visualise slide scanned images of 6 H&E-stained sections from each knee, ≥75 µm apart, and arthritis severity scores were assigned at 0.25 increments to indicate the observed extent of synovial lining hyperplasia (0-3), immune cell infiltration (0-3), cellular exudate (0-3), and erosions at the patellofemoral joint margins (0-3). To determine the erosion score, separate scores were assigned to medial and lateral sides of the patella and femoral condyles, and the average erosion score was calculated for each section. For each parameter, the average score per knee was calculated, and scores of the 4 parameters were then summed to give an overall arthritis score (0-12). For TRAP+ cell quantification, cells were counted along the medial and lateral femoral periosteum, or endosteum of the femoral epiphysis, and the average per knee was calculated. Cell counting was performed using ImageJ 1.47v, QuPath 0.1.2 or Zen 2.1 Lite software and normalised to length of synovium analysed. For mouse IHC analysis, the average DAB intensity of 2 or 3 sections per mouse was calculated in a 600 µm-length area along the medial patellofemoral synovium using QuPath 0.2.0

software.[4] Replicate sections were stained on separate occasions, and within each staining experiment, data were normalised to the average DAB intensity of the control group. For human IHC analysis, the average DAB intensity of 1-5 sections per donor was measured in 200-400 µm-length areas of quiescent and hyperplastic synovium using QuPath 0.2.0 software.[4] Hyperplastic areas were recognised by the more roundish shape and large nuclei of RA-SF, as previously described.[5] Sections were stained in two separate batches and data were normalised to the average DAB intensity of quiescent areas. For the *Yap* KO experiments, researchers were fully blinded to genotypes throughout all experiments, outcome assessments, and data analyses.

Micro-CT analysis

4% PFA-fixed mouse knee joints were scanned using a Skyscan 1072 micro-CT system (Bruker) with the following settings: 40x magnification, 50 kV, 0.5 mm Al filter, 200 µA source current, 6.3 sec exposure time, 0.68° rotation step over 180 degrees. Images were reconstructed using Nrecon 2.0.4.0 and visualised using Dataviewer 1.5.6.2, CtVol 2.0 and CtVox 3.3 (Bruker). Analysis was carried out using Ctan v1.13. To accurately segment the bone structures across the whole dataset, a threshold was set at 85-255 for the attenuation histogram and regions of interest (ROIs) were drawn around the trabecular bone of the tibial epiphysis every 5 sections over a range of 105 sections to define the volume of interest (VOI) for each limb.

Cell isolation, sorting, and culture

Cells were isolated from mouse knee joints by digestion with 1 mg/ml collagenase type IV (Sigma) as described,[2] according to a protocol optimised for synovial cell isolation.[6] Cells were left unsorted, or were sorted according to Tom fluorescence in conjunction with CD45-APC and Pdgfrα-BV421 immunostaining (Supplementary Table 6), using an Influx Cell Sorter (BD Biosciences) as previously

Symons RA, *et al. Ann Rheum Dis* 2022; 781:214–224. doi: 10.1136/annrheumdis-2021-220875

described.[2] For *in vitro* experiments, cells were expanded in high-glucose Dulbecco's Modified Eagle Medium (DMEM; Lonza) supplemented with 10% foetal bovine serum (FBS; ThermoFisher Scientific), 100 units ml^{-1} of penicillin and 0.1 mg ml^{-1} streptomycin (Sigma). C3H10T1/2 clone 8 mouse embryonic fibroblasts and RAW264.7 mouse macrophages were purchased from the American Type Culture Collection. C3H10T1/2 cells were maintained in alpha Minimum Essential Medium (αMEM) supplemented with 4 mM L-glutamine and 10% FBS. RAW264.7 cells were cultured in low-glucose DMEM with 2mM L-glutamine and 10% FBS.

Immunophenotyping of cells

Cells were stained with antibodies listed in Supplementary Table 6 and analysed by flow cytometry. Data were acquired on a BD LSRII or Fortessa flow cytometer and analysed using FlowJo v10 software. Unstained and single-labelled cells or antibody-labelled CompBeads (BD Biosciences) were used to set compensation, and gating was informed by Fluorescence-Minus-One (FMO) controls and cells stained with isotype negative control antibodies, as appropriate. Staining with Fixable Viability Dye eFluor 455UV (eBioscience, cat. no. 65-0868-18) or eFluor 780 (eBioscience, cat. no. 65-0865-14) was used to exclude dead cells as indicated.

Gene overexpression, silencing and KO *in vitro*

p2xFLAG-hYAP1-S127A was a gift from Marius Sudol (Addgene plasmid #17790; http://n2t.net/addgene:17790; RRID:Addgene_17790).[7] p3xFLAG-mSnail was a gift from Celeste Nelson (Addgene plasmid #34583; http://n2t.net/addgene:34583; RRID:Addgene_34583).[8] pCMV-Tag2A plasmid (Stratagene) was used as negative control. DsiRNA sequences (Supplementary Table 8) were obtained from IDT (Integrated DNA Technologies). The universal negative control 1 (NC1) from IDT was used. Transient transfections were performed using TransIT-X2 (Mirusbio) on cells plated at a

density of 10,000 cells/cm^2 and left to adhere for 24 h prior to transfection according to manufacturer's protocol. Medium was replaced 24 h after transfection, and cells were used for assays after a further 24 h. For *Yap* KO *in vitro*, cells isolated from *Yap*$^{fl/fl}$;*Tom* mice, or *Tom* mice as controls, were plated at a density of 1,600 cells/cm^2 and transduced with lentivirus encoding Cre recombinase. The LV-Cre plasmid was a gift from Inder Verma (Addgene plasmid # 12106; http://n2t.net/addgene:12106; RRID:Addgene_12016) and lentivirus was produced as described above. After 9 days, lentivirally-transduced cells were used to assess the effect of *Yap* KO on proliferation.

RNA extraction, cDNA synthesis, and quantitative PCR

RNA was extracted using TRIZOL reagent (Invitrogen), miRNEasy/RNEasy Micro kits (Qiagen) or PicoPure RNA Isolation Kit (ThermoFisher) using standard protocols, and quantified using a NanoDrop ND-1000 spectrophotometer (Labtech). cDNA was synthesised from up to 1 µg of total RNA using random hexamer primers and SuperScript IV Reverse Transcriptase (Invitrogen), according to the manufacturer's instructions. Quantitative PCR (qPCR) was performed with a Roche LightCycler 480 using SYBR Green Master (Roche). Primers were designed using Primer-BLAST (NCBI) and are listed in Supplementary Table 9. Amplification of a single product of correct size was confirmed by agarose gel electrophoresis and/or melting curve analysis. Relative concentrations were quantified by ΔΔCt or standard curve method, and normalised to expression of HPRT or GAPDH.

Cell invasion

Cells were serum-starved overnight and seeded at 20,000 cells/well in the upper chamber of a 6.5 mm diameter, 8 µm pore, transwell plate (Corning) pre-coated with Matrigel Growth Factor Reduced Basement Membrane Matrix (Corning) at a concentration of 0.4 mg/ml. The upper and lower

Symons RA, *et al. Ann Rheum Dis* 2022; 781:214–224. doi: 10.1136/annrheumdis-2021-220875

compartments were filled with serum-free DMEM and DMEM containing 20% FBS, respectively. Cells were incubated for 24 h, fixed with 4% PFA, and stained with 0.5% crystal violet solution. Cotton swabs were used to remove non-invasive cells from the upper chambers. Image tiling on a Zeiss LSM710 inverted confocal microscope was used to scan each transwell, and invasive cells were counted in 5 standard pre-determined regions and the average was calculated.

Cell proliferation

Cre-transduced *Yap*$^{fl/fl}$;*Tom* or *Tom* SF were plated at a density of 4,200 cells/cm^2, left to adhere overnight, and then incubated with EdU (10 µM) for 16 hours. EdU incorporation into replicating DNA was detected using an Alexa Fluor 647 Click-iT EdU detection kit (ThermoFisher Scientific). Flow cytometry analysis was performed on a BD LSRII flow cytometer (BD Biosciences) and the percentage of EdU-positive cells quantified using FlowJo v10 software (Tree Star).

Yap-Tead GFP reporter assay

The 8xGTIIC-luciferase vector was a gift from Stefano Piccolo (Addgene plasmid #34615; http://n2t.net/addgene:34615; RRID:Addgene_34615).[9] To generate stable fluorescent Yap reporter cells, Tead DNA-binding sequences were sub-cloned from the 8xGTIIC-luciferase vector into the XbaI/ClaI site of a pGreenFire lentiviral reporter vector (System Biosciences). The strategy allows control of copGFP expression by Yap-Tead binding upstream of an mCMV promoter. Lentivirus was produced in HEK293T cells using jetPRIME (Polyplus-transfection), and packaging plasmids pMDLg/pRRE (Addgene plasmid #12251; http://n2t.net/addgene:12251; RRID:Addgene_12251) and pRSV-Rev (Addgene plasmid #12253; http://n2t.net/addgene:12253; RRID:Addgene_12253), and envelope plasmid pMD2.G (Addgene plasmid #12259; http://n2t.net/addgene:12259; RRID:Addgene_12259), all a gift from Didier Trono. C3H10T1/2 cells were transduced with the

pGreenFire Yap reporter lentivirus or pGreenFire empty vector lentivirus. Monoclonal Yap reporter cell lines were isolated using cloning cylinders. Stable Yap reporter cell lines were validated by transient transfection with the p2xFLAGhYAP1 plasmid (Addgene plasmid #17791; http://n2t.net/addgene:17791; RRID:Addgene_17791) to overexpress Yap using jetPRIME (Polyplus-transfection), according to manufacturer's protocol. One Yap reporter cell line was selected for further experiments. Yap reporter and empty vector control cells were seeded at 2,000 cells/cm^2 in normal growth media and allowed to attach for 24 h before overnight serum starvation followed by 48 h of stimulation under serum-free conditions, as indicated. GFP fluorescence was detected using a BD Fortessa flow cytometer and analysed using FlowJo v10. GFP fluorescence was normalised to the empty vector control cells for each condition.

Protein expression and western blotting

C3H10T1/2 Yap-Tead reporter cells were seeded at 20,000 cells/cm^2 in high-glucose DMEM (10% FBS, 4 mM L-glutamine) and allowed to attach for 24 h, before serum starvation for 2 h and stimulation under serum-free conditions as indicated. Cells were lysed in PBS containing 0.1% sodium dodecyl sulphate (w/v), 0.5% sodium deoxycholate (w/v), 1% Igepal, 2% (v/v) protease inhibitor cocktail (Sigma, P8340) and 1% (v/v) each of phosphatase inhibitor cocktails (Sigma, P0044 and P5726). Protein was quantified via bicinchoninic acid (BCA) assay (ThermoScientific), and equal amounts of total protein were separated by SDS-PAGE. After semi-dry transfer to FL-PVDF membrane (Merck), blots were probed with antibodies listed in Supplementary Table 7, and antibody binding was detected using a LiCor Odyssey Imaging System.

Symons RA, *et al. Ann Rheum Dis* 2022; 781:214–224. doi: 10.1136/annrheumdis-2021-220875

Proximity ligation assay

Cells were fixed in 4% PFA for 10 min, washed 3 times in PBS and incubated in 1% BSA in TBS-T for 45 mins, before incubation with antibodies against Yap (0.07 µg/ml) and Snail (5 µg/ml) (Supplementary Table 5) overnight at 4°C. Yap-Snail complexes were detected by proximity ligation assay using the Duolink In Situ PLA Probe Anti-Goat PLUS (Sigma), Duolink In Situ PLA Probe Anti-Rabbit MINUS (Sigma) and Duolink In Situ Detection Reagents FarRed (Sigma), according to the manufacturer's instructions, followed by staining and mounting with Duolink In Situ Mounting Medium with DAPI (Sigma). For each well, 10 images from random areas were acquired on a Zeiss LSM710 confocal microscope, and the number of Yap-Snail complexes per cell was counted (average of 8 cells per image counted).

Statistical analysis

All data points on graphs, and n-numbers in text, indicate individual human donors or mice, or independent experiments. SigmaPlot v13 or v14 and GraphPad Prism v5 software were used for statistical analysis. Tests used to determine statistical significance ($p<0.05$) are indicated in figure legends. Normality and equal variances were tested in Sigmaplot using the Shapiro-Wilk and Brown-Forsythe tests, respectively. Ordinal data were analysed using a non-parametric Mann-Whitney U test. Log-transformation was used to equalise variance prior to statistical testing as indicated. One *Yap* WT AIA knee was accidentally damaged during sample processing, and another showed patellar dislocation upon histological examination. These two mice were excluded. A Grubb's outlier test was used to identify and exclude 1 major outlier from the WT group of the *Yap* cKO experiments, with an arthritis score of 1.108 and a z-value of 3.137 ($p<0.05$). The Pearson's test was used for correlation analysis.

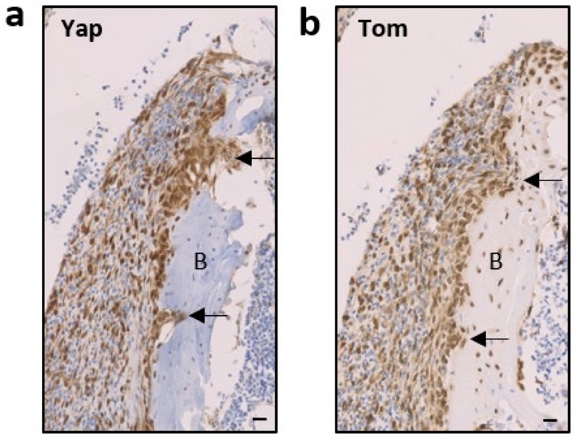

Supplementary Figure 1. Yap is expressed by Tom+ *Gdf5*-lineage cells at sites of erosion. Detection of Yap (**a**) and Tom (**b**) by IHC in near-adjacent sections of a knee from a *Gdf5-Cre*;*Tom* mouse 6 days after AIA induction (n=6; 2 males, 4 females, 10-13 weeks, from 2 experiments). Arrows indicate Yap+ and Tom+ cells, respectively, at sites of erosion along the periosteal surface close to the joint margin. Scale bars indicate 20 µm. B: bone.

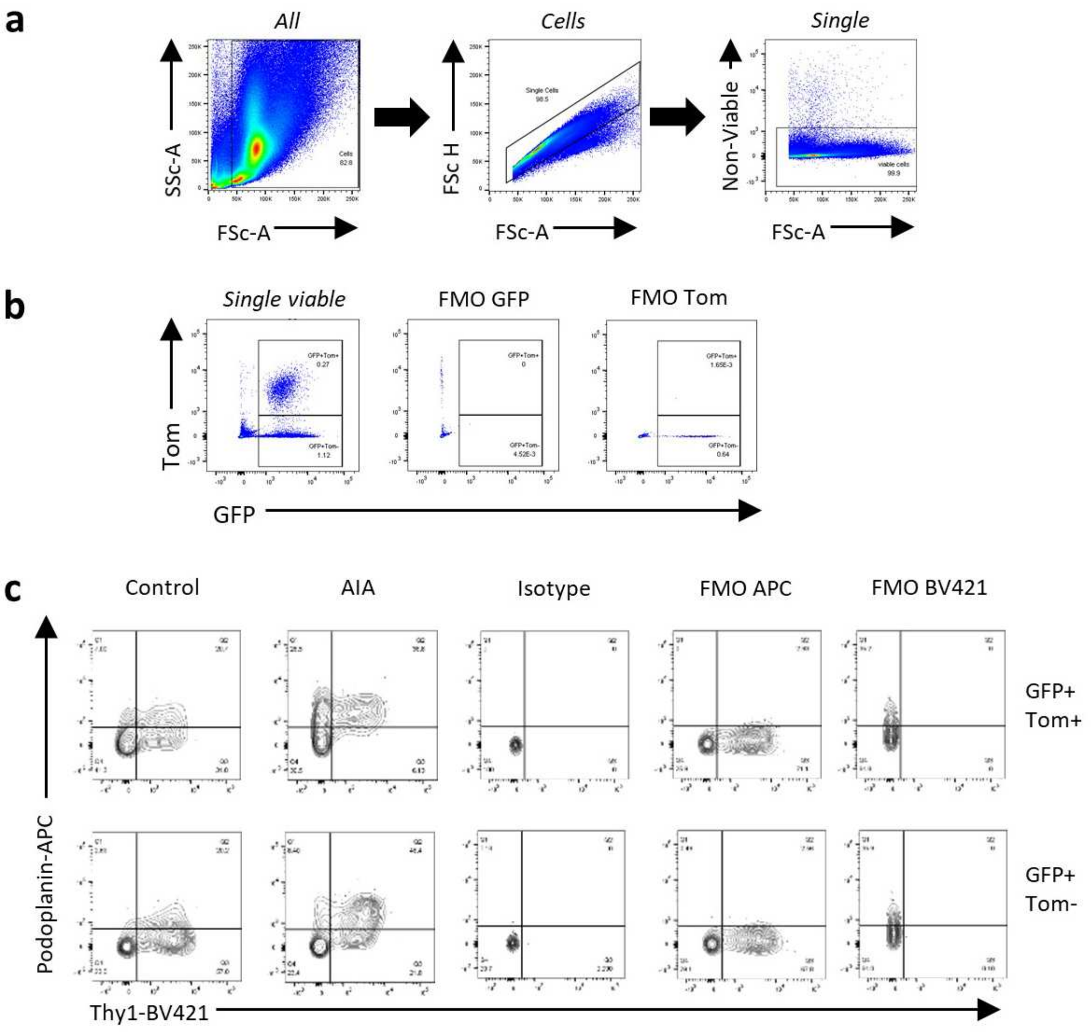

Supplementary Figure 2. Extended data Figure 4a,b. Freshly isolated cells from knees of adult *Gdf5-Cre*;*Tom;Pdgfrα-H2BGFP* mice 6 days after AIA induction were analysed by flow cytometry (n=6; 5 males, 1 female, 11-14 weeks, pooled data from 2 experiments). (**a**) Gating strategy to identify single live cells. Erythrocytes and debris were gated out based on Forward and Side Scatter profile. Doublets and aggregates were excluded based on Forward Scatter parameters. Dead cells were excluded based on viability dye staining. (**b**) Gating strategy to identify, within single live cells, the GFP+Tom+ and GFP+Tom- cells. Gates were set using fluorescence-minus-one (FMO) controls, namely cells from a *Gdf5-Cre*;*Tom* mouse (FMO GFP) or *Pdgfrα-H2BGFP* mouse (FMO Tom). (**c**) Representative flow cytometry plots for the analysis of Podoplanin and Thy1 expression within the GFP+Tom+ and GFP+Tom- cell populations. AIA: Cells isolated from AIA knee; Control: Cells isolated from contralateral control knee. Gates were set using cells stained with isotype negative control antibodies (isotype), and cells not stained with the respective antibody, which served as fluorescence-minus-one (FMO) controls.

Symons RA, *et al. Ann Rheum Dis* 2022; 781:214–224. doi: 10.1136/annrheumdis-2021-220875

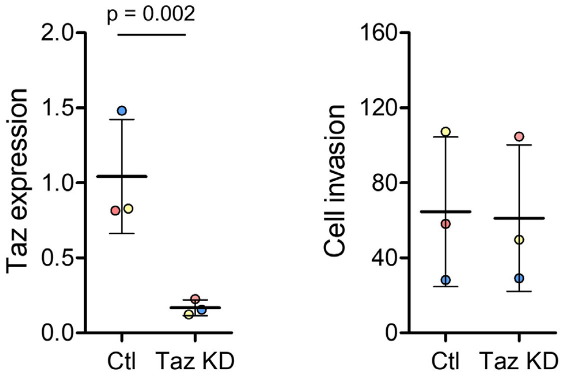

Supplementary Figure 3. Lack of effect of Taz knockdown (KD) on AIA-SF invasiveness through matrigel in a transwell assay. Taz expression was analysed by qRT-PCR to confirm efficient Taz KD. Dots are colour-coded to indicate 3 experiments using independent SF cultures from 3 AIA mice. P-value indicates significance based on two-tailed paired Student's t–test.

Symons RA, *et al. Ann Rheum Dis* 2022; 781:214–224. doi: 10.1136/annrheumdis-2021-220875

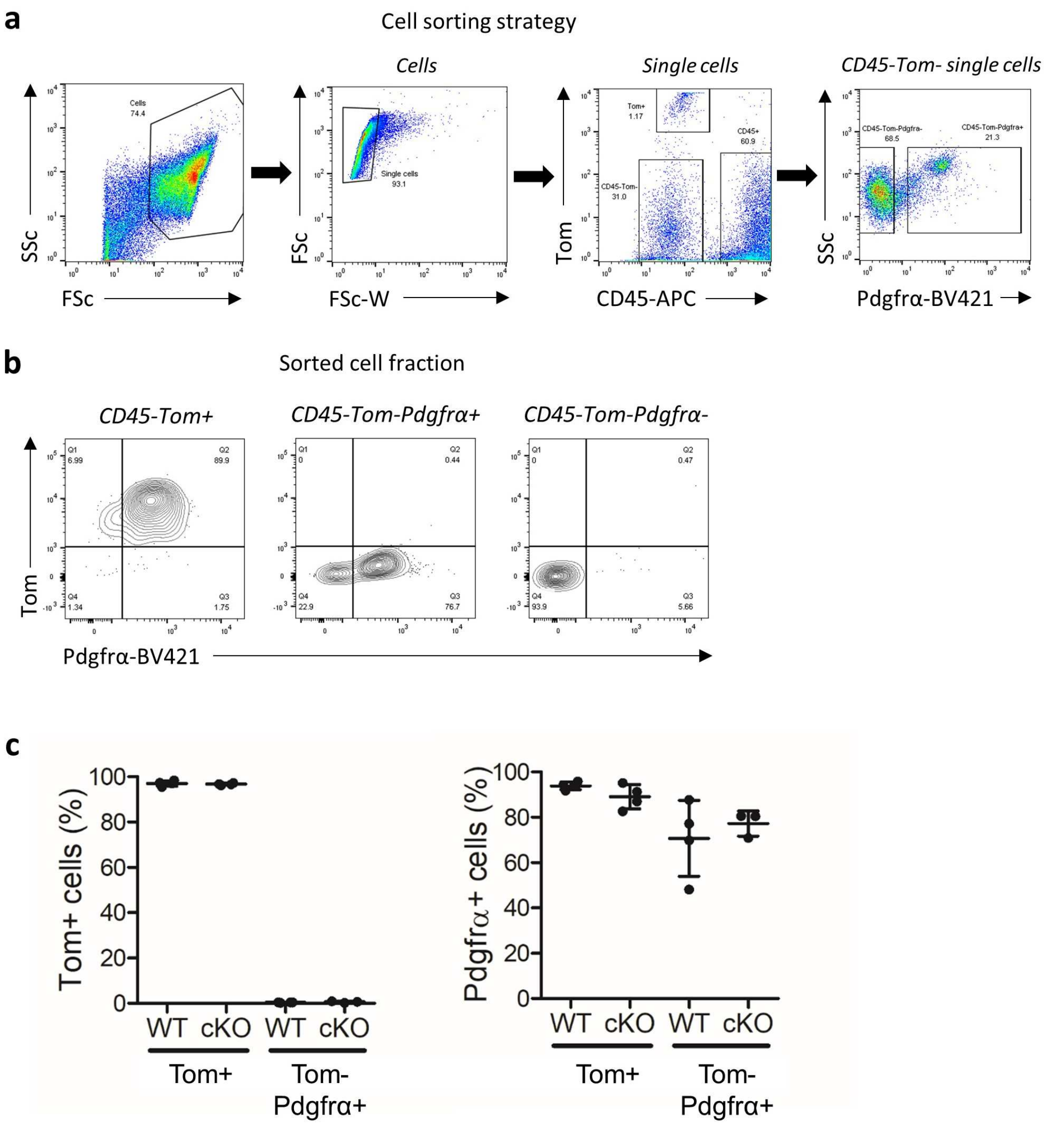

Supplementary Figure 4. Freshly isolated cells from knees of adult *Gdf5-Cre*;*Tom*;*Yap*$^{WT/WT}$ (*Yap* WT, n=4) and *Gdf5-Cre*;*Tom*;*Yap*$^{fl/fl}$ mice (*Yap* cKO, n=4), 7 days after AIA induction, were sorted on a BD Influx cell sorter. (**a**) Gating strategy for FACS performed on a BD Influx cell sorter. Debris was gated out based on Forward (FSc) and Side Scatter (SSc) profile. Doublets and aggregates were excluded based on FSc parameters. *Gdf5*-lineage cells were identified by Tom fluorescence and haematopoietic cells by CD45-APC immunostaining. Non-*Gdf5*-lineage fibroblasts were identified from within the Tom-CD45- population by Pdgfrα-BV421 immunostaining. Gates are drawn for illustration purposes. (**b**,**c**) Flow cytometry analysis of aliquots of sorted cells on an LSRII flow cytometer to determine purity. Lines and error bars indicate mean ± SD (n=3-4; 1 *Yap* cKO Tom-Pdgfrα+ sorted sample was excluded from analysis due to very low cell yield). See Supplementary Table 2 for mouse genotypes, sex and age.

Symons RA, *et al. Ann Rheum Dis* 2022; 781:214–224. doi: 10.1136/annrheumdis-2021-220875

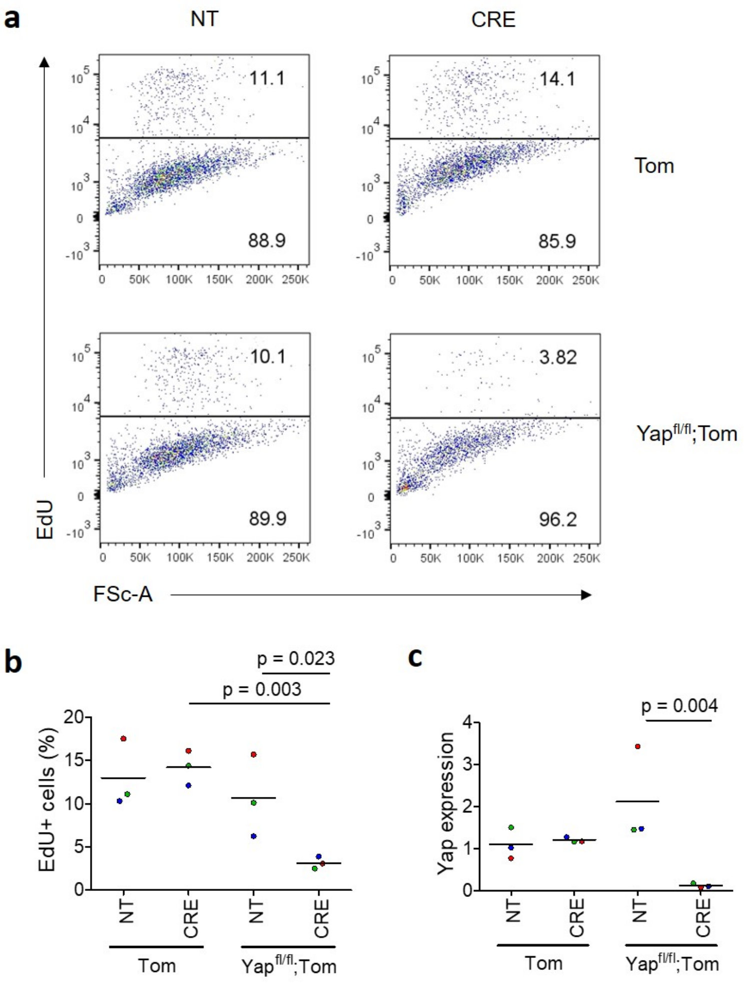

Supplementary Figure 5. Effect of *Yap* KO on mouse SF proliferation *in vitro*. Cultured cells from knees of adult *Yap$^{WT/WT}$;Tom (Tom)* or *Yap$^{fl/fl}$;Tom* mice, transduced to express *Cre,* were incubated with EdU and then analysed by flow cytometry. (**a**) Representative flow cytometry plots showing EdU incorporation indicative of proliferation. (**b**) Percentage of EdU+ cells. (**c**) Confirmation of *Yap* KO by qRT-PCR. Colour-coding indicates 3 experiments using SF cultures from different mice. P-values indicate results of two-way ANOVA with Tukey's post-hoc test.

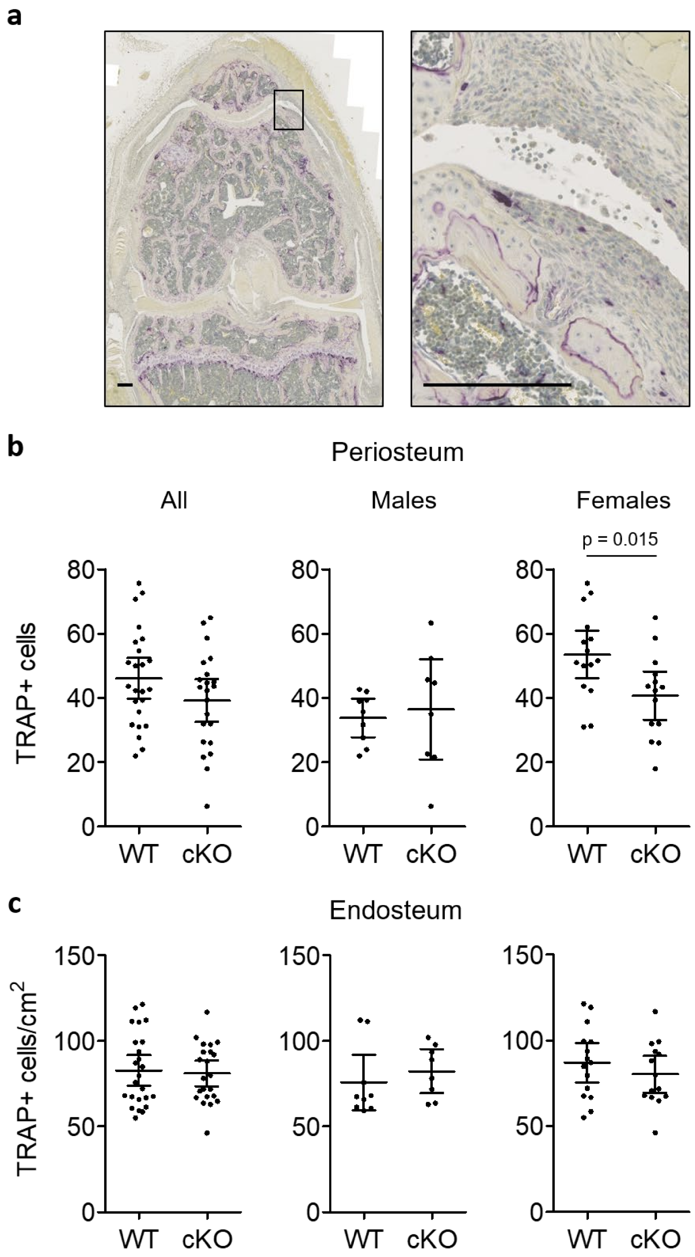

Supplementary Figure 6. Extended data Figure 5h. Histochemical analysis of TRAP+ cells in *Yap* WT and *Yap* cKO mice. AIA was induced in *Yap* WT or *Yap* cKO mice. Histological quantification of TRAP+ cells in tissue sections was performed 9 days later. (**a**) TRAP+ cells were counted along the medial and lateral femoral periosteum. Boxed area in image on the left is shown at higher magnification on the right. Scale bars: 200 µm. (**b**) Average number of TRAP+ cells along the femoral periosteal surface per section. (**c**) Average number of TRAP+ cells along the endosteum of the femoral epiphysis, relative to total area of femoral epiphysis. Dots represent individual mice with mean ± 95% CI indicated (WT: n=24 [M=9; F=15]; cKO: n=22 [M=8; F=14]). P-value indicates statistical significance based on unpaired two-tailed t test. See Supplementary Table 2 for mouse genotypes, sex and age.

Symons RA, *et al. Ann Rheum Dis* 2022; 781:214–224. doi: 10.1136/annrheumdis-2021-220875

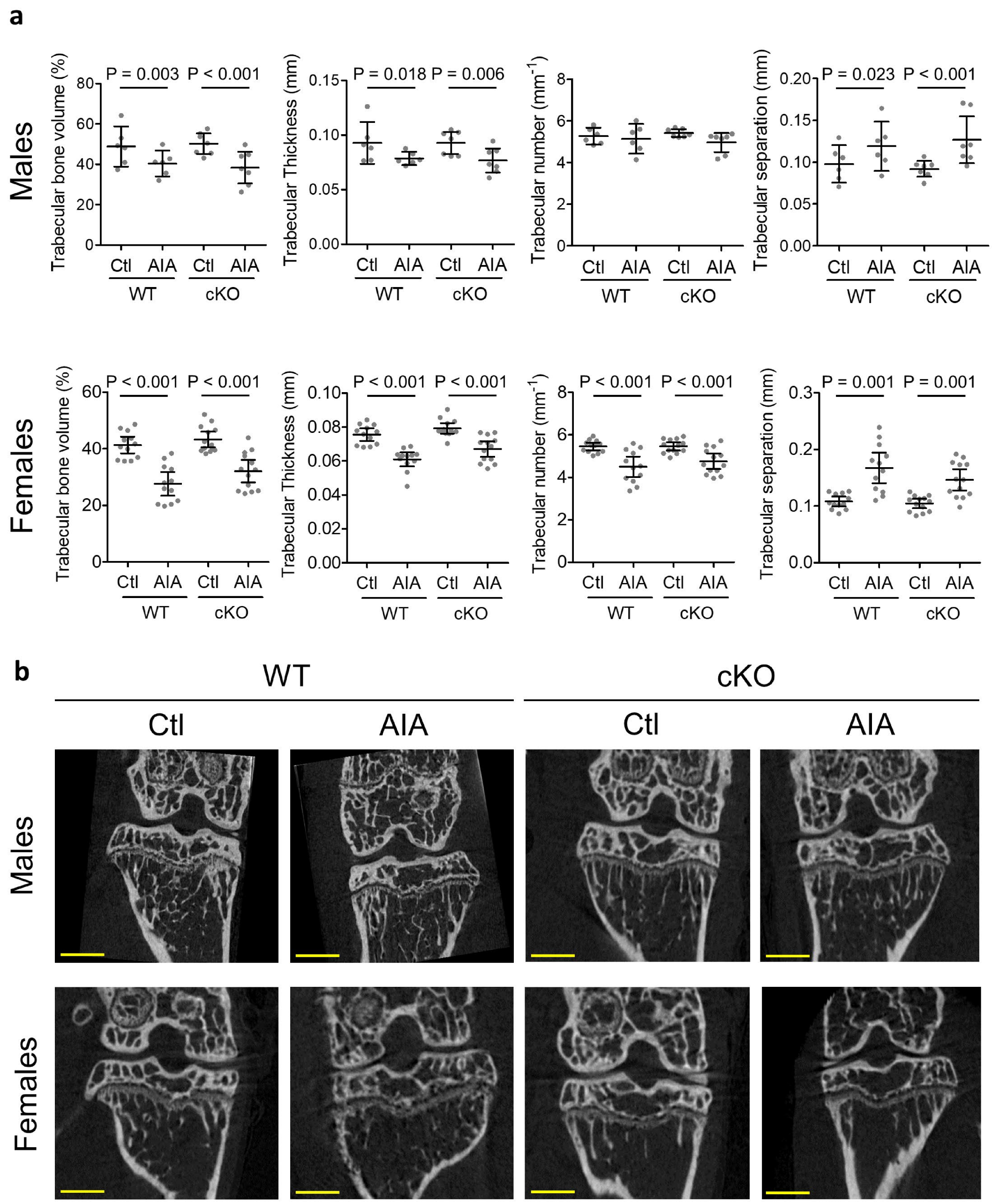

Supplementary Figure 7. Micro-CT analysis of trabecular bone in the tibial epiphysis of *Yap* WT and *Yap* cKO mice. AIA was induced in one knee of *Yap* WT or *Yap* cKO mice, with the contralateral knee serving as control. Micro-CT analysis was performed 9 days after arthritis induction. (**a**) Data are shown as mean ± 95% CI with dots indicating individual mice. P-values indicate statistical significance based on repeated measures two-way ANOVA with Tukey's post-test. (**b**) Representative micro-CT images. Scale bars: 1 mm. See Supplementary Table 2 for mouse genotypes, sex and age.

Symons RA, *et al. Ann Rheum Dis* 2022; 781:214–224. doi: 10.1136/annrheumdis-2021-220875

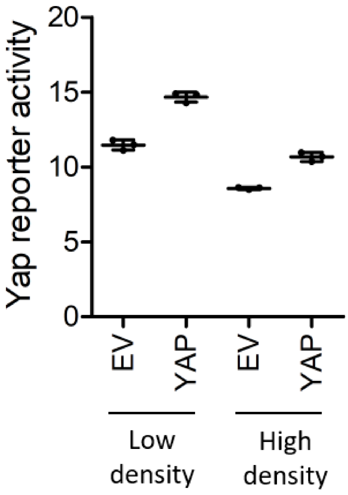

Supplementary Figure 8. Validation of Yap-Tead GFP reporter cell line. C3H10T1/2 Yap-Tead GFP reporter cells, or cells transduced with empty lentiviral vector (control cells), were transfected with either *YAP* or empty vector (EV) plasmid. After 24 h, cells were seeded in 12-well plates at $5x10^3$ (low density) or $25x10^3$ (high density) cells per well and GFP expression was measured by flow cytometry after a further 24 h. Data are expressed as geo mean GFP fluorescence intensity relative to the control cells for each condition. n = 3 technical replicates. Results demonstrate responsiveness of the Yap-Tead GFP reporter to cell density as well as *YAP* overexpression.

Symons RA, *et al. Ann Rheum Dis* 2022; 781:214–224. doi: 10.1136/annrheumdis-2021-220875

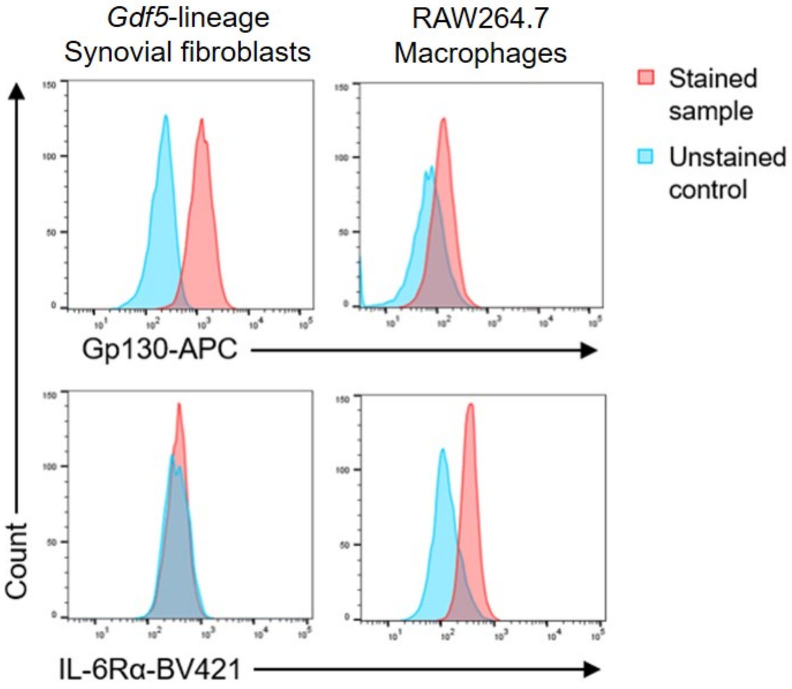

Supplementary Figure 9. *Gdf5*-lineage SF express the Gp130 subunit, but not the IL6Rα subunit, of the IL6 receptor. Cell surface expression of Gp130 and IL6Rα in cultured *Gdf5*-lineage SF isolated from knees of adult *Gdf5-Cre;Tom;Pdgfrα-H2BGFP* mice was detected by flow cytometry, with RAW264.7 macrophages analysed as a positive control. Representative histograms show fluorescence intensity of IL6Rα-BV421 and Gp130-APC stained (red) and unstained (blue) cells. Representative of cells from n = 3 mice.

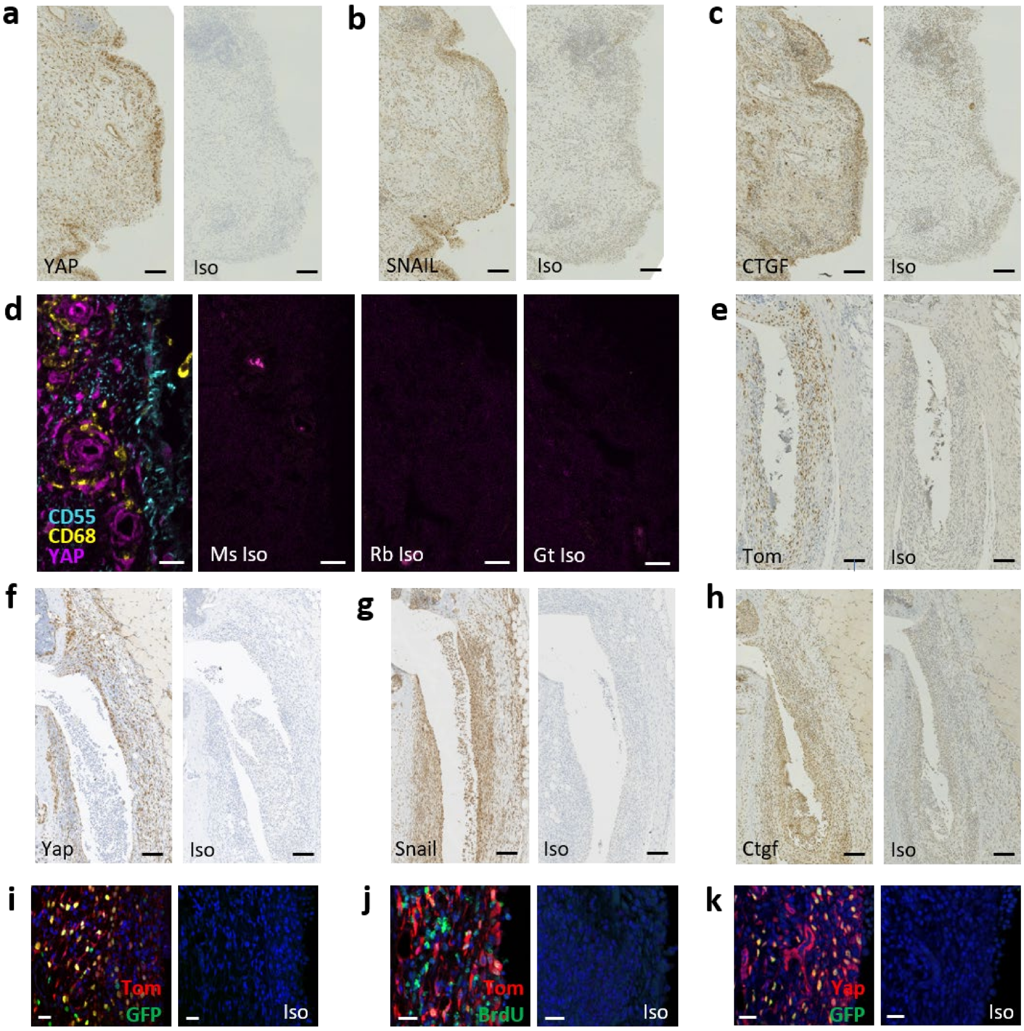

Supplementary Figure 10. Validation of immunostainings. (**a**-**c**) Extended data for Fig. 1a, showing detection of (**a**) YAP, (**b**) SNAIL, and (**c**) CTGF in human RA synovium, with matched isotype control antibody stainings (IgG). (**d**) Extended data for Fig. 1b, showing detection of YAP (magenta), the lining SF marker CD55 (cyan) and the macrophage marker CD68 (yellow) in human RA synovium, with matched isotype control antibody stainings. Different tissue sections were stained with each of the isotype control antibodies. Ms IgG: Mouse isotype control IgG, corresponding to CD68 antibody. Rb IgG: Rabbit isotype control IgG, corresponding to YAP antibody. Gt IgG: Goat isotype control IgG, corresponding to CD55 antibody. (**e**) Extended data for Fig. 5e, showing detection of Tom in mouse AIA synovium, with matched isotype control antibody staining (IgG). (**f**-**h**) Extended data for Figs. 2a-c, 5a, and 6a, showing detection of (**f**) Yap, (**g**) Snail, and (**h**) Ctgf in mouse AIA synovium, with matched isotype control antibody stainings (IgG). (**i**-**k**) Extended data for Fig. 3c,d,f and 5f, showing co-detection of (**i**) Tom (red) and GFP (green), (**j**) Tom (red) and BrdU (green), and (**k**) Yap (red) and GFP (green) in mouse AIA synovium, with matched isotype control antibody stainings (IgG and IgY). All isotype negative control antibody stainings were performed within the same immunostaining experiment and on tissue sections from the same sample as the fully stained sections shown in this figure. Scale bars: 100 µm for IHC and 20 µm for IF images.

Symons RA, *et al. Ann Rheum Dis* 2022; 781:214–224. doi: 10.1136/annrheumdis-2021-220875

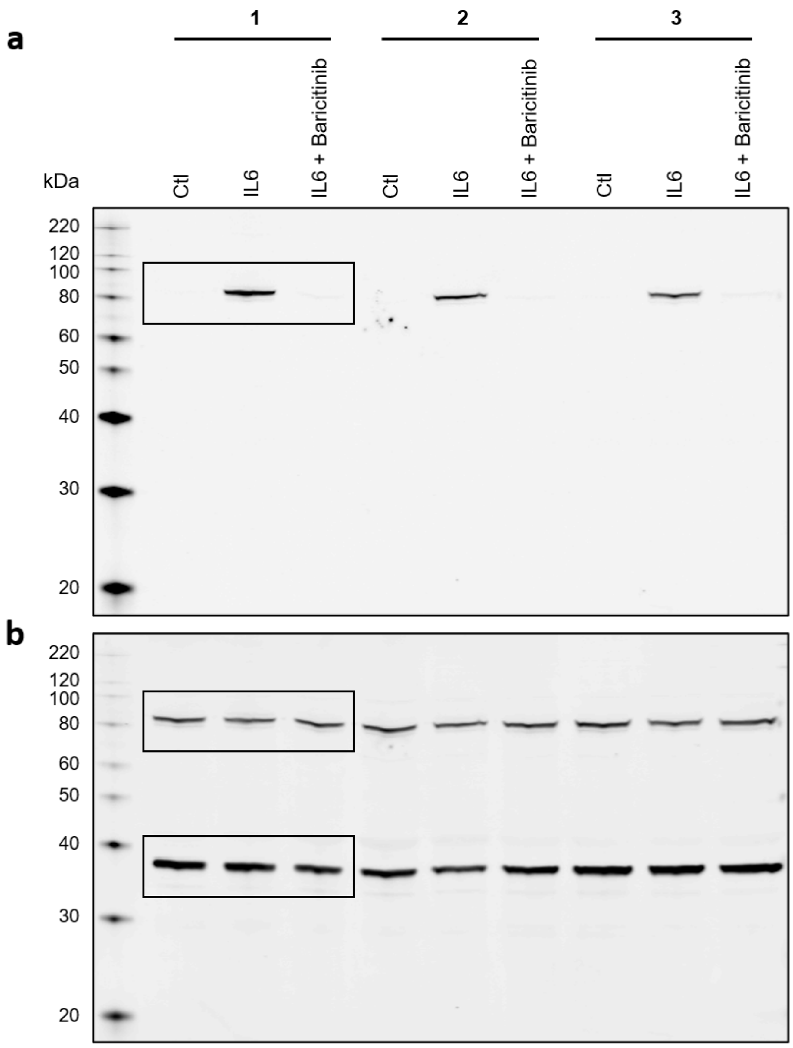

Supplementary Figure 11. Extended data for Figure 7c. Uncropped Western blot images showing Stat3 phosphorylation in Yap-Tead GFP reporter cells. C3H10T1/2 Yap-Tead reporter cells were pre-treated for 1 h with either baricitinib (10 µM) or vehicle (0.1% DMSO). IL6/sIL6R (140 ng/ml) was then added as indicated for 30 min. The blot was imaged to show (**a**) pStat3 detected at 800 nm, and (**b**) total Stat3 and Gapdh detected at 700 nm, on a LI-COR Odyssey Infrared Imager. Boxed areas correspond to cropped regions shown in Figure 7c.

Supplementary Table 1. Transgenic mouse lines.

Short name	Full name	Source	Ref.
Gdf5-Cre	Tg(*Gdf5-cre-ALPP*)1Kng	D Kingsley (Stanford)	[1]
TdTomato	B6.Cg-Gt(*ROSA*)26Sortm14(*CAG-tdTomato*)Hze/J	JAX, stock no. 6774	[10]
Pdgfrα-H2BGFP	B6.129S4-*Pdgfra*tm11(*EGFP*)Sor/J	JAX, stock no. 7669	[11]
Pdgfrα-CreER	B6N.Cg-Tg(*Pdgfra-cre/ERT*)467Dbe/J	JAX, stock no. 18280	[12]
Confetti	STOCK Gt(*ROSA*)26Sortm1(*CAG*-Brainbow2.1)Cle/J	JAX, stock no. 13731	[13]
Yap1-flox	*Yap1*tm1.1Fcam	F Camargo (Harvard)	[14]

Supplementary Table 2. *Yap* cKO mice.

Figure	Mice	Genotype [n]	Sex [n]	Age at end in weeks [range]	Notes [n]
5a,c-e,h, Suppl. Fig 6,7	*Yap* WT	*Gdf5-Cre*;*Yap*$^{WT/WT}$;*Tom* [21]<br>*Gdf5-Cre*;*Yap*$^{WT/WT}$ [1]<br>*Yap*$^{fl/fl}$;*Tom* [2]	M [9]; F [15]	14.1 [11.4-15.9]	Leaky mice [3] and mice without *Tom* [1] or *Gdf5-Cre* [2] were excluded from Tom analysis.
	Yap cKO	*Gdf5-Cre*;*Yap*$^{fl/fl}$;*Tom* [22]	M [8]; F [14]	14.3 [11.6-16.0]	
5b,g Suppl. Fig 4	*Yap* WT	*Gdf5-Cre*;*Yap*$^{WT/WT}$;*Tom* [4]	M [2]; F [2]	14.3 [13.3-16.1]	
	Yap cKO	*Gdf5-Cre*;*Yap*$^{fl/fl}$;*Tom* [4]	M [2]; F [2]	13.9 [11.6-15.7]	Tom-Pdgfrα+ sorted sample [1] had low cell yield and was excluded from analysis (Fig. 5b, Suppl. Fig 4).
5f	*Yap* cKO	*Gdf5-Cre*;*Yap*$^{fl/fl}$;*Tom* [8]	M [7]; F [1]	13.1 [9.9-14.6]	

Supplementary Table 3. *Yap* ciKO mice.

Figure	Mice	Genotype [n]	Sex [n]	Age at end in weeks [range]
6	*Yap* WT	*Yap*$^{fl/fl}$ [9]	M [4]; F [5]	15.2 [15.0-15.4]
	Yap ciKO	*Pdgfrα-CreER*;*Yap*$^{fl/fl}$ [6]	M [3]; F [3]	15.2 [15.0-15.3]

Supplementary Table 4. RA patient information.

Patient	Age	Sex	Procedure	Joint	Disease duration	ACPA	RF	Usage
1	68	M	US-guided biopsy	Knee	16 months	Positive	Positive	qPCR
2	58	F	US-guided biopsy	Knee	24 months	Positive	Positive	qPCR
3	56	M	US-guided biopsy	Knee	90 months	Negative	Negative	qPCR
4	57	F	US-guided biopsy	Knee	6 months	Positive	Positive	qPCR
5	57	F	US-guided biopsy	Knee	48 months	Negative	Negative	qPCR
6	55	F	US-guided biopsy	Knee	6 months	Negative	Positive	qPCR
7	61	F	US-guided biopsy	Knee	55 months	Negative	Positive	qPCR
8	54	F	US-guided biopsy	Knee	11 months	Negative	Negative	qPCR
9	60	F	US-guided biopsy	Knee	120 months	Negative	Negative	qPCR
10	60	F	US-guided biopsy	Knee	9 months	Positive	Positive	qPCR, IHC
11	63	F	US-guided biopsy	Knee	120 months	Positive	Positive	IHC
12	62	F	US-guided biopsy	Knee	12 months	Negative	Negative	IHC
13	52	F	Arthroplasty	Knee	9 years	Negative	Negative	IHC, IF, CC
14	83	M	Arthroplasty	Knee	18 years	n/a	Positive	IF
15	69	F	Arthroplasty	Knee	6 years	Negative	Positive	IHC, IF
16	54	M	Arthroplasty	Knee	12 years	n/a	n/a	IHC, CC
17	66	F	Arthroplasty	Knee	16 years	n/a	Positive	IHC, IF, CC

ACPA: anti-citrullinated protein antibodies; RF: Rheumatoid Factor; US: ultrasound; n/a: information not available; qPCR: quantitative RT-PCR; IHC: immunohistochemistry; IF: immunofluorescence staining; CC: cell culture.

Symons RA, *et al. Ann Rheum Dis* 2022; 781:214–224. doi: 10.1136/annrheumdis-2021-220875

Supplementary Table 5. Antibodies for immunohistochemistry and immunofluorescence staining.

Antibody	Clone	Manufacturer	Cat. No.	Conjugation
Yap	D8H1X	Cell Signaling	14074	unconjugated
Snail	polyclonal	Abcam	ab53519	unconjugated
Ctgf	polyclonal	Abcam	ab6992	unconjugated
mCherry (detects Tom)	polyclonal	Sicgen	ab0081	unconjugated
mCherry (detects Tom)	polyclonal	Novus Biologicals	NBP2-25158SS	unconjugated
RFP (detects Tom)	polyclonal	Rockland	600-401-379	unconjugated
GFP	polyclonal	Abcam	ab6556	unconjugated
GFP	polyclonal	Abcam	ab13970	unconjugated
BrdU	BU1/75 (ICR1)	Abcam	ab6326	unconjugated
CD55	polyclonal	R&D Systems	AF2009	unconjugated
CD68	KP1	eBioscience	14-0688-82	unconjugated
Rabbit IgG isotype control	DA1E	Cell Signaling	3900	unconjugated
Rabbit IgG isotype control	polyclonal	R&D Systems	AB-105-C	unconjugated
Goat IgG isotype control	polyclonal	R&D Systems	AB-108-C	unconjugated
Mouse IgG1 isotype control	G3A1	Cell Signaling	5415	unconjugated
Rat IgG1 isotype control	43414	R&D Systems	MAB005	unconjugated
Chicken IgY isotype control	polyclonal	R&D Systems	AB-101-C	unconjugated
Donkey anti-goat IgG	polyclonal	Abcam	ab150129	Alexa Fluor 488
Donkey anti-rabbit IgG	polyclonal	Abcam	ab150068	Alexa Fluor 594
Donkey anti-rat IgG	polyclonal	Abcam	ab150156	Alexa Fluor 594
Horse anti-goat	polyclonal	Vector	BA-9500	biotinylated
Goat anti-rabbit	polyclonal	Vector	BA-1000	biotinylated

Supplementary Table 6. Antibodies for flow cytometry.

Antibody	Clone	Manufacturer	Cat. No.	Conjugation
CD90	OX-7	BD Biosciences	563770	BV421
Podoplanin	8.1.1	Biolegend	127410	APC
Pdgfrα	APA5	BD Horizon	562774	BV421
CD45	30-F11	BD Pharmingen	559865	APC
IL-6Rα	D7715A7	BD Biosciences	740038	BV421
Gp130	KPG130	eBioscience	17-1302-82	APC

Supplementary Table 7. Antibodies for western blotting.

Antibody	Clone	Manufacturer	Cat. No.	Conjugation
pStat3	D3A7	Cell Signaling	#9145	unconjugated
Stat3	124H6	Cell Signaling	#9139	unconjugated
Gapdh	6C5	Abcam	ab8245	unconjugated
Donkey anti-mouse	polyclonal	LI-COR	926-68072	IRDye 680RD
Goat anti-rabbit	polyclonal	LI-COR	926-32211	IRDye 800CW

Symons RA, *et al. Ann Rheum Dis* 2022; 781:214–224. doi: 10.1136/annrheumdis-2021-220875

Supplementary Table 8. DsiRNA sequences.

Name	Sequence 5'-3'
Mouse Yap DsiRNA #1 sense	rArUrCrUrUrCrUrGrGrUrCrArArArGrArUrArCrUrUrCrUTA
Mouse Yap DsiRNA #1 antisense	rUrArArGrArArGrUrArUrCrUrUrUrGrArCrCrArGrArArGrArUrGrU
Mouse Yap DsiRNA #2 sense	rCrCrArCrCrArArGrCrUrArGrArUrArArArGrArArArGrCTT
Mouse Yap DsiRNA #2 antisense	rArArGrCrUrUrUrCrUrUrUrArUrCrUrArGrCrUrUrGrGrUrGrGrCrA
Mouse Taz DsiRNA sense	rGrArUrArCrUrUrCrCrUrUrArArUrCrArCrArUrArGrArGAA
Mouse Taz DsiRNA antisense	rUrUrCrUrCrUrArUrGrUrGrArUrUrArArGrGrArArGrUrArUrCrUrC
Human YAP DsiRNA sense	rGrGrUrCrArGrArGrArUrArCrUrUrCrUrUrArArArUrCrACA
Human YAP DsiRNA antisense	rUrGrUrGrArUrUrUrArArGrArArGrUrArUrCrUrCrUrGrArCrCrArG
Human TAZ DsiRNA sense	rGrGrUrArCrUrUrCrCrUrCrArArUrCrArCrArUrArGrArAAA
Human TAZ DsiRNA antisense	rUrUrUrUrCrUrArUrGrUrGrArUrUrGrArGrGrArArGrUrArCrCrUrC
Negative control 1 DsiRNA sense	rCrGrUrUrArArUrCrGrCrGrUrArUrArArUrArCrGrCrGrUAT
Negative control 1 DsiRNA antisense	rArUrArCrGrCrGrUrArUrUrArUrArCrGrCrGrArUrUrArArCrGrArC

Supplementary Table 9. qPCR primer sequences.

Gene	Sequence 5'-3'	
	Forward primer	Reverse primer
Mouse Yap	GCCCGACTCCTTCTTCAAG	GAGTGAGCTCGAACATGCT
Mouse Taz	AGCTCAGATCCTTTCCTCAATG	ACCTGTATCCATCTCGTCCAT
Mouse Gapdh	TGGATTTGGACGCATTGGTC	TTTGCACTGGTACGTGTTGAT
Mouse Hprt	CAAACTTTGCTTTCCCTGGT	CAACAAAGTCTGGCCTGTATC
Mouse Mmp3	TCTGGGCTATACGAGGGCACGAGG	TGGCAGCATCGATCTTCTTCACGG
Mouse Mmp9	CCATGCACTGGGCTTAGATCAT	CAGATACTGGATGCCGTCTATGTC
Mouse Mmp12	GGAGCTCACGGAGACTTCAACT	CCTTGAATACCAGGTCCAGGATA
Mouse Mmp13	AAGATGTGGAGTGCCTGATG	AAGGCCTTCTCCACTTCAGA
Mouse Mmp14	TAAGCACTGGGTGTTTGACGAA	CCCTCGGCCAAGCTCCT
Mouse IL6	GAGGATACCACTCCCAACAGACC	AAGTGCATCATCGTTGTTCATACA
Mouse IL33	TCCTTGCTTGGCAGTATCCA	TGCTCAATGTGTCAACAGACG
Mouse IL34	ACTCAGAGTGGCCAACATCACAAG	ATTGAGACTCACCAAGACCCACAG
Human YAP	CGCTCTTCAACGCCGTCA	AGTACTGGCCTGTCGGGAGT
Human TAZ	TATCCCAGCCAAATCTCGTG	TTCTGCTGGCTCAGGGTACT
Human SNAIL	AATCGGAAGCCTAACTACAGCG	GTCCCAGATGAGCATTGGCA
Human CTGF	GTTTGGCCCAGACCCAACT	GGAACAGGCGCTCCACTCT
Human GP130	AGGACCAAAGATGCCTCAAC	GAATGAAGATCGGGTGGATG
Human GAPDH	AACAGCGACACCCACTCCTC	CATACCAGGAAATGAGCTTGACAA

References

1 Rountree RB, Schoor M, Chen H, *et al.* BMP receptor signaling is required for postnatal maintenance of articular cartilage. *PLoS Biol* 2004;**2**.

2 Roelofs AJ, Zupan J, Riemen AHK, *et al.* Joint morphogenetic cells in the adult mammalian synovium. *Nat Commun* 2017;**8**:15040.

3 Roelofs AJ, De Bari C. Immunostaining of Skeletal Tissues. In: *Methods in molecular biology (Clifton, N.J.)*. Humana Press 2019. 437–50.

4 Bankhead P, Loughrey MB, Fernández JA, *et al.* QuPath: Open source software for digital pathology image analysis. *Sci Rep* 2017;**7**:1–7.

5 Ospelt C, Neidhart M, Gay RE, *et al*. Synovial activation in rheumatoid arthritis. *Front Biosci* 2004;**9**:2323–34.

6 Armaka M, Gkretsi V, Kontoyiannis D, *et al.* A standardized protocol for the isolation and culture of normal and arthritogenic murine synovial fibroblasts. *Nat. Protoc. Exchange 2009.*

7 Komuro A, Nagai M, Navin NE, *et al.* WW domain-containing protein YAP associates with ErbB-4 and acts as a co-transcriptional activator for the carboxyl-terminal fragment of ErbB-4 that translocates to the nucleus. *J Biol Chem* 2003;**278**:33334–41.

8 Lee K, Gjorevski N, Boghaert E, *et al.* Snail1, Snail2, and E47 promote mammary epithelial branching morphogenesis. *EMBO J* 2011;**30**:2662–74.

9 Dupont S, Morsut L, Aragona M, *et al.* Role of YAP/TAZ in mechanotransduction. *Nature* 2011;**474**:179–83.

10 Madisen L, Zwingman TA, Sunkin SM, *et al.* A robust and high-throughput Cre reporting and characterization system for the whole mouse brain. *Nat Neurosci* 2010;**13**:133–40.

11 Hamilton TG, Klinghoffer RA, Corrin PD, *et al.* Evolutionary divergence of platelet-derived growth factor alpha receptor signaling mechanisms. *Mol Cell Biol* 2003;**23**:4013–25.

12 Kang SH, Fukaya M, Yang JK, *et al.* NG2+ CNS glial progenitors remain committed to the oligodendrocyte lineage in postnatal life and following neurodegeneration. *Neuron* 2010;**68**:668–81.

13 Snippert HJ, van der Flier LG, Sato T, *et al.* Intestinal Crypt Homeostasis Results from Neutral Competition between Symmetrically Dividing Lgr5 Stem Cells. *Cell* 2010;**143**:134–44.

14 Schlegelmilch K, Mohseni M, Kirak O, *et al.* Yap1 acts downstream of alpha-catenin to control epidermal proliferation. *Cell* 2011;**144**:782–95.
